# Microwave brain imaging system to detect brain tumor using metamaterial loaded stacked antenna array

**DOI:** 10.1038/s41598-022-20944-8

**Published:** 2022-10-01

**Authors:** Amran Hossain, Mohammad Tariqul Islam, Gan Kok Beng, Saad Bin Abul Kashem, Mohamed S. Soliman, Norbahiah Misran, Muhammad E. H. Chowdhury

**Affiliations:** 1 Centre for Advanced Electronic and Communication Engineering, Department of Electrical, Electronic and Systems Engineering, Faculty of Engineering and Built Environment, Universiti Kebangsaan Malaysia, 43600 Bangi, Malaysia; 2 Department of Computer Science and Engineering, Dhaka University of Engineering and Technology, Gazipur, Gazipur, 1707 Bangladesh; 3 Department of Electrical, Electronic and Systems Engineering, Faculty of Engineering and Built Environment, Universiti Kebangsaan Malaysia, 43600 Bangi, Malaysia; 4 Department of Computer Science, AFG College with the University of Aberdeen, Doha, Qatar; 5 Department of Electrical Engineering, College of Engineering, Taif University, P.O. Box 11099, Taif, 21944 Saudi Arabia; 6 Department of Electrical Engineering, Faculty of Energy Engineering, Aswan University, Aswan, 81528 Egypt; 7 Department of Electrical Engineering, Qatar University, Doha, Qatar

**Keywords:** Head and neck cancer, Brain imaging, Sensors and biosensors, Biological physics

## Abstract

In this paper, proposes a microwave brain imaging system to detect brain tumors using a metamaterial (MTM) loaded three-dimensional (3D) stacked wideband antenna array. The antenna is comprised of metamaterial-loaded with three substrate layers, including two air gaps. One 1 × 4 MTM array element is used in the top layer and middle layer, and one 3 × 2 MTM array element is used in the bottom layer. The MTM array elements in layers are utilized to enhance the performance concerning antenna’s efficiency, bandwidth, realized gain, radiation directionality in free space and near the head model. The antenna is fabricated on cost-effective Rogers RT5880 and RO4350B substrate, and the optimized dimension of the antenna is 50 × 40 × 8.66 mm^3^. The measured results show that the antenna has a fractional bandwidth of 79.20% (1.37–3.16 GHz), 93% radiation efficiency, 98% high fidelity factor, 6.67 dBi gain, and adequate field penetration in the head tissue with a maximum of 0.0018 W/kg specific absorption rate. In addition, a 3D realistic tissue-mimicking head phantom is fabricated and measured to verify the performance of the antenna. Later, a nine-antenna array-based microwave brain imaging (MBI) system is implemented and investigated by using phantom model. After that, the scattering parameters are collected, analyzed, and then processed by the Iteratively Corrected delay-multiply-and-sum algorithm to detect and reconstruct the brain tumor images. The imaging results demonstrated that the implemented MBI system can successfully detect the target benign and malignant tumors with their locations inside the brain.

## Introduction

Globally, brain-related diseases such as brain tumors are an enormous burden for people and health care systems^1^. A brain tumor is one of the serious reasons for death because it damages the main tissues of the brain. A brain tumor is a mass growth of abnormal cells that form inside the head and transforms into brain cancer. Brain cancer can be a threat to human life and critically affect the longevity of life. Brain cancer possibility is growing over time due to the uncontrolled growth of the brain tumors and it is the 9th main cause of death for the human being^2^. The Brain tumor is primarily classified into two types: (i) benign tumor and (ii) malignant tumor^3^. The number of brain tumor cases around the globe is growing at a frightening rate. According to the National Brain Tumor Society (NBTS), 88,970 people in the USA live with a primary brain tumor diagnosis in 2022, where 63,040 are affected by benign tumors, and 25,930 are affected by malignant tumors tumor^4^. The benign tumor is non-cancerous cells having homogeneous structure with a regular shape. It does not invade neighbouring tissue or spread to other parts of the body. The malignant tumor is a cancerous cell with a heterogeneous structure with an irregular shape. The benign tumors grow very slowly, whereas malignant tumors grow uncontrollably. The death rate increases because of invasive characteristics of the tumors, but initial detection, monitoring, and proper investigation can reduce the death rate and increase the survival rate. Nowadays, different categories of imaging technologies: computed tomography (CT) scanning, X-ray screening, magnetic resonance imaging (MRI), biopsy, positron emission tomography (PET), and ultrasound screening are used to diagnose brain tumors in modern medical healthcare facilities^5–7^. These existing imaging modalities help physicians and radiologists detect brain tumors and other health-related diseases. The major drawbacks of these imaging technologies are growing cancerous risk because of high dose radiation, dangerous for pregnant women and old patients, high ionizing with brain cells, expensive, the risk for pacemaker and implantable cardioverter patients, time-consuming, and less susceptibility^8–13^. Microwave imaging research has been growing and showed excellent attention to the researchers for medical applications due to significant characteristics such as: cost-effective with a low profile, non-ionizing radioactivity, non-invasive, risk-free ionization with the tissues, low powered penetration capability, and safe for human body^13–19^. Recently, a microwave brain imaging (MBI) system has been utilized to identify the brain abnormalities such as brain tumors, cancer, stroke, and internal hemorrhage in the brain. The MBI system consists of an antenna array, mechanical devices, and an image processing unit. The antenna is an essential piece of equipment, and its characteristics are a significant factor in producing the desired image. A single antenna transmits the microwave signals towards the region of interest, and receiver antenna(s) receive the backscattering signals. Different antennas have been offered to develop an MBI system to detect brain tumors. A fern antipodal Vivaldi antenna was proposed in^20^ to detect brain tumor. The dimension of the antenna was 150 × 150 mm^2^, and the operating frequency was 1–3.3 GHz. The authors in^20^ only implemented a simulation-based imaging system. They did not implement any experimental system, and no SAR (specific absorption rate) analysis was presented. A brick-shaped antenna was designed for the MBI system to detect brain abnormality^21^. The bandwidth of the antenna was only 400 MHz, with low gain. The authors experimented with the system by using two antennas and tested by the conventional head phantom. They presented only measured data and did not provide a reconstructed image sample. A wideband W-shaped slot-loaded U-shaped rectangular patch antenna was proposed in^22^ to identify the tumor in the microwave imaging system. The antenna’s operating band was 1.40–2.52 GHz with 3.5 dBi gain. The measured SAR was 0.30 W/kg, and radiation efficiency was 95%. The authors designed an imaging system using a nine-antenna array and simulated the system using CST software. Then collected simulated data for tumor characterization, but they did not exhibit reconstruction tumor image for validation.

An MBI system using six ultrawideband (UWB) antenna array was presented in^23^. In the MBI system, a semi-circular head model was utilized by simulation technique to detect brain tumor. Authors have used a symplectic finite-difference time-domain algorithm to reconstruct the brain images. The approach presented a reconstructed image with a single tumor object, but they used simulated data to reconstruct the image. Also, the approach showed the irregular shaped tumor object instead of regular shaped tumor and it is able to detect the tumor nearby the skull. In^24^, the MBI system was proposed to identify the single target tumor object. The proposed system used a twelve-metamaterial-loaded spear-shaped wideband antenna array to reconstruct the brain tumor image. The dimension of the antenna was 32 × 24 mm^2^, and the operating band was 0.75–1.6 GHz. The system was tested by using the SAM (Specific Anthropomorphic Mannequin) head phantom model, including bone, brain, and blood clot as a tumor. However, the system produced the image with the target tumor. But the reconstructed image was noisy, and tumor location identification is very difficult due to low gain and radiation directivity. A single EBG-based patch antenna for brain tumor detection in the MBI system was presented in^25^. The spherical simulated head phantom model verified the system via scattering parameters, and no experimental analysis was explained. The reconstructed image was blurry due to the low gain of the antenna and achieved a maximum SAR of 0.922 W/kg. The system cannot detect the tumor in-depth location, and difficult to localize the tumor in a blurry image. A twelve GCPW (ground coplanar waveguide) wideband antenna array-based head imaging system was discussed in^26^ to diagnose a brain tumor. The antenna’s dimension was 50 × 44 mm^2^, and the functioning frequency band was 1.70–3.71 GHz with 5.65 dBi gain. The authors performed the system by utilizing a Hugo head model and analyzed the simulated data, but no imaging result was presented. The maximum SAR was 0.00233 W/Kg. In^27^, a three-dimensional (3D) directive wideband antenna was proposed to identify brain tumor in the human brain. The overall dimension was 40 × 25 × 10 mm^3^ and obtained operating band was 1.67–1.74 GHz with 5.2 dBi gain. The simulated system was performed by two antenna setups and reconstructed the brain images, but the generated image was noisy. A 3D slot-rotated wideband antenna was reported in^28^ to identify the brain tumor. The antenna's dimension, operating band, and gain are 50 × 34 × 25, 1.41–3.57 GHz, and 2.6 dBi, respectively. The imaging system consists of a large platform, a rotated SAM head phantom model with a single antenna for scanning the whole area. The reconstructed image showed healthy image and a malignant tumor-based image but localize the target tumor is difficult for non-expert physicians due to blurry image and no SAR calculation are presented. In^29^, proposed a thirteen-antenna array based experimental head imaging system for identifying the tumor object. The system was examined by using a tissue simulating fluid as a head model and reconstructed the images. The system detected a single tumor object as an abnormality of the brain. A unidirectional 3D wideband antenna was presented for detecting tumor in the microwave head imaging system^30^. The system was designed by two antenna array setups as a simulation model and used simulated phantom model. Later, the raster scanning process collects scattering data and reconstructs brain images to detect the malignant tumor. A portable head imaging system with a 16 antenna array was proposed for the diagnosis of a brain tumor^31^. The system reconstructed images with a single target tumor, but the equipment is costly and needs a large platform for imaging. A metamaterial based multi-layered UWB patch antenna was proposed for microwave imaging (MWI) to detect tumor^32^. The antenna achieved maximum gain of 5 dBi with an operating bandwidth of 7 GHz. However, the antenna was investigated using a simulated phantom model to identify the tumor. No experimental analysis was presented in the study. Another UWB patch antenna was proposed in for MWI to identify the tumor cell in breast^33^. The antenna was compact dimension with high bandwidth, but the gain of the antenna is only 3 dBi. However, the antenna was investigated using a simulated phantom model to identify the tumor cell. The authors did not present experimental analysis in the paper. A low-cost MWI system using side slotted tapered slot antenna was presented in^34^ to identify the tumor cells. The authors in^34^, used the iteratively corrected delay-and-sum (IC-DAS) algorithm for reconstructing the tumor images. The antenna was investigated by employing a tumor-based phantom model. However, the reconstructed images were blurry and noisy.

It is observed that the reported MBI systems used different types of antennas for identifying the target tumor object. The reconstructed images from the systems are low resolution based, noisy, and blurry. As a result, tumor identification by non-expert physicians and radiologists can be difficult. These problems have occurred due to deficiency of antenna’s radiation directivity, low gain, incapability of signal penetration, and high SAR. Also, the systems are examined to detect only a single tumor. In most cases, both benign and malignant tumors can be formed in the brain. In those cases, the existing systems can be failed to detect both tumors. Therefore, there is a demand to implement an experimental MBI system by utilizing a significant wideband antenna that can detect benign and malignant tumor in the brain in the early stage. So, an efficient wideband antenna is required in an MBI system that has high gain and efficiency, directional radiation capability for signal penetration, high bandwidth, and low SAR. In this research, we developed an MBI system by utilizing a 3D stacked antenna array that can detect both tumors. The novelties of this research are as follows:

1. According to authors best knowledge, this is the first research work, where a new metamaterial-loaded three-dimensional (3D) stacked wideband antenna is proposed for detecting the two tumors (i.e., benign and malignant tumor) in microwave brain imaging system.
2. The metamaterial (MTM) array is utilized in staked layers of the antenna to enhance the bandwidth, realized gain, efficiency, and capability of radiation directivity for adequate signal penetration to the brain. In addition, the new designed MTM unit cell has reduced the SAR and improved the fidelity factor that ensures less distortion for receiving the backscattered signals.
3. Implemented a nine-antenna array-based portable microwave brain imaging system that can reconstruct noiseless and high-resolution brain images for detecting the benign and malignant tumors.
4. Fabricated a six-layered tissue-mimicking head phantom model, including tumors, to verify the system performance.
5. Compared the outcomes of the implemented system with the state-of-the-art MBI system to ensure better performance of the proposed antenna.

The remaining part of the research is organized as follows: The proposed antenna design and analysis are explained in “Metamaterial loaded 3D stacked antenna design” section. Performance analysis of the proposed antenna is discussed in “Antenna performance analysis” section. “Sensitivity measurements of the head model” section discussed the sensitivity analysis of the head model by the antenna. A detailed experimental investigation of the microwave brain imaging system, phantom fabrication and measurement, and image reconstruction results are explained in “Microwave brain imaging results and discussion” section and concluded in “Conclusion” section.

## Metamaterial loaded 3D stacked antenna design

### Antenna structure and MTM unit cell analysis

**Figure 1:**
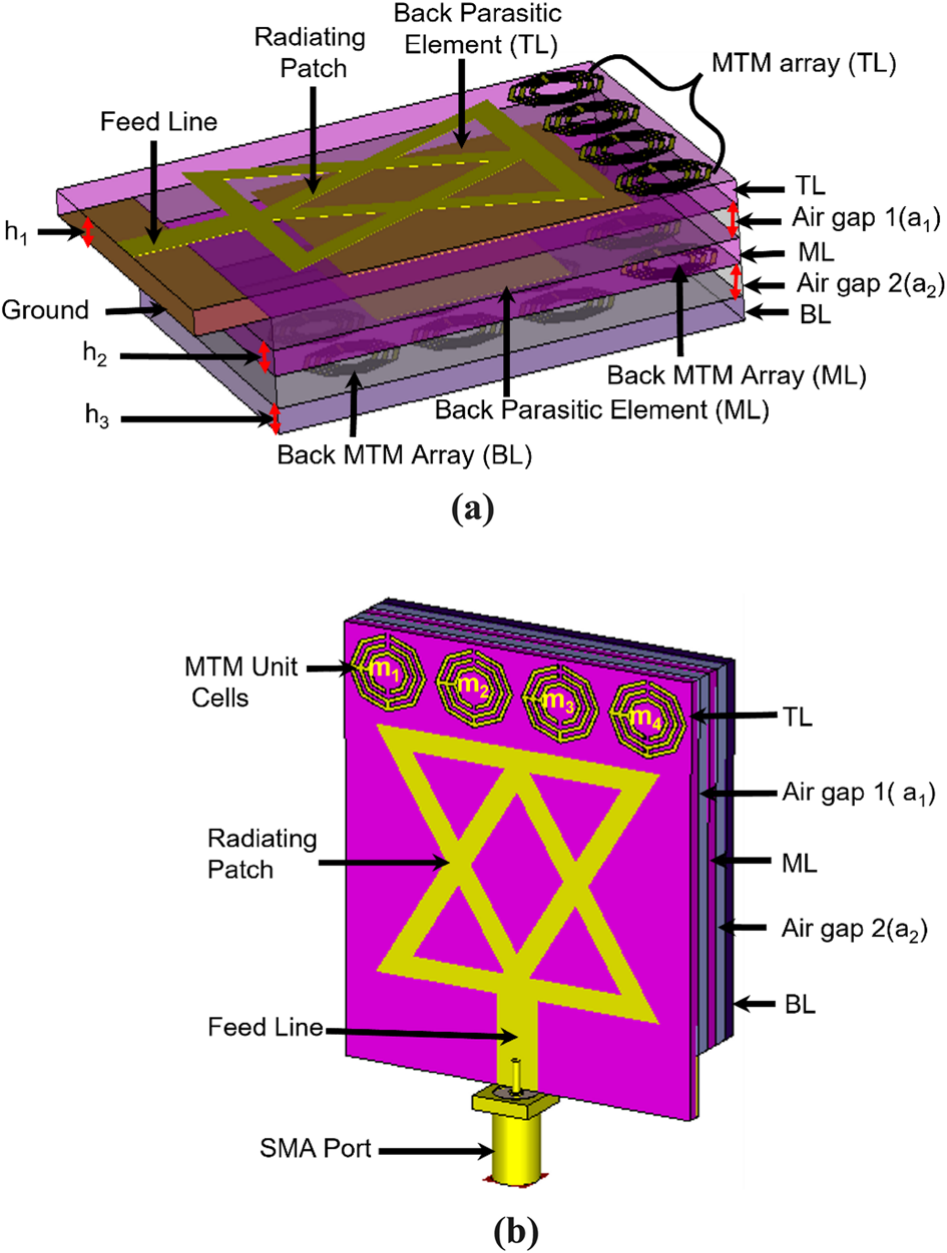
Proposed 3D stacked antenna: (**a**) Overall layout; (**b**) 3D model.

**Figure 2:**
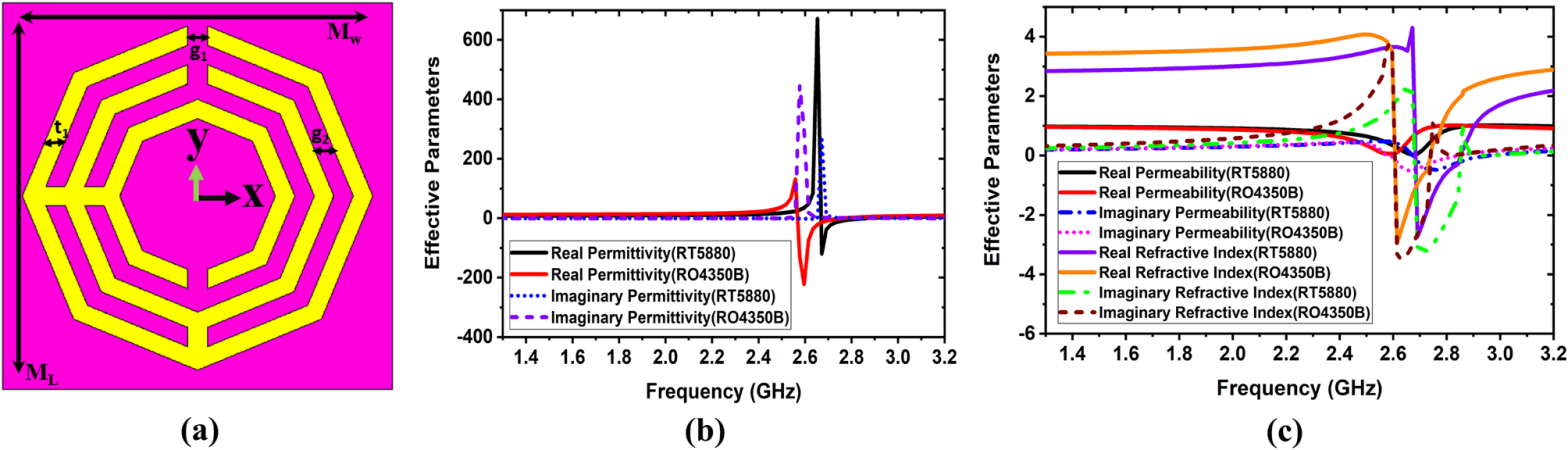
Spider net-shaped metamaterial unit cell: (**a**) MTM unit cell geometry; (**b**, **c**) Extracted effective parameters. The dimensions of the unit cell (mm): M_w_ = 9, M_L_ = 9, g_1_ = g_2_ = t_1_ = 0.5.

The schematic diagram of the metamaterial (MTM) loaded 3D stacked antenna’s structure is depicted in Fig. 1. The antenna consists of three layers: Top Layer (TL), Middle Layer (ML), and Bottom Layer (BL). The MTM array unit cells are used in three layers. The 2 mm thickness of the air gap is considered between TL and ML, ML and BL. The Rogers RT5880 substrate (Relative permittivity ε_r_ = 2.2, loss tangent δ = 0.0009, thickness h_1_ = h_2_ = 1.575 mm) material is used in TL and ML, whereas the Rogers RO4350B substrate (Relative permittivity ε_r_ = 3.66, loss tangent δ = 0.0037, thickness h_3_ = 1.524 mm) material is used in BL. The ML is attached with the TL, and the BL is attached with the ML by using a thickness of 2 mm double-sided foam tape. The main radiating patch and feed line are designed on the TL. A 50Ω SMA port is connected to the feeding line that feds the signal to the antenna. The antenna’s feed line width is responsible for 50Ω impedance matching. The spider net-shaped MTM array is employed on the TL, ML, and BL to enhance the antenna’s performance. A 1 × 4 MTM array (m_1_ to m_4_) is used on the top layer and backside of the middle layer, and a 3 × 2 MTM array (m_1_ to m_6_) is used on the backside the bottom layer to enhance the radiation directionality, gain, and bandwidth of the antenna. The proposed antenna’s overall 3D layer-based stacked structure layout is illustrated in Fig. 1a, and its model is illustrated in Fig. 1b. The three layers of the antenna, including two air gaps (a_1_ and a_2_) made it a three-dimensional (3D) stacked antenna structure. The stacked structure is responsible for the high performance of the antenna. The spider net-shaped MTM unit cell’s geometric structure and simulated effective parameters are depicted in Fig. 2. The MTM unit cell is depicted in Fig. 2a. The simulation is performed by the FDS (Frequency-domain solver) based simulator CST (Computer Simulator Tool) software to design, investigate, and analyze the cell's properties. For achieving the optimal electromagnetic field distribution, the MTM unit cell is positioned between the + Z axis and -Z axis waveguide ports and powered towards the Z-axis by the wave.

The PEC (perfect electric conducting) and PMC (perfect magnetic conducting) walls are imposed on ± x and ± y directions of the unit cell to emulate the periodic nature of the cell^35^. The unit cell is simulated by using both RT5880 and RO4350B substrate materials and analysis of the effective parameters. The Nicolson-Ross-weir (NRW) method^36^ is utilized to extract the effective parameters (real and imaginary) from the scattering parameters as follows:

1 $$Permittivity\,(\varepsilon_{r} ) = \frac{2}{{jk_{0} d}} \times \left( {\frac{{1 - S_{21} + S_{11} }}{{1 + S_{21} - S_{11} }}} \right)$$

2 $$Permeability\,(\mu_{r} ) = \frac{{j2S_{11} }}{{jk_{0} d}} + \mu {}_{0}$$

3 $$Refractive\,Index\,(\eta_{r} ) = \frac{2}{{jk_{0} d}}\sqrt {\frac{{(S_{21} - 1)^{2} - S_{11}^{2} }}{{(S_{21} + 1)^{2} - S_{11}^{2} }}}$$

4 $$k_{0} = \omega \sqrt {\mu_{0} \varepsilon_{0} }$$

where *S*_11_ denotes the reflection coefficient, *S*_21_ denotes the transmission coefficient, *k*_0_ denotes the wave number, and *d* denotes the substrate thickness. The frequency range for the MTM in the proposed antenna is 1.30–3.20 GHz. The real and imaginary permittivity of the unit cell is shown in Fig. 2b. In Fig. 2b, the −ve real permittivity frequency ranges are 2.65–2.86 GHz for RT5880 and 2.57–2.74 GHz for RO4350B, whereas the −ve imaginary permittivity frequency ranges are 1.30–2.65, 2.97–3.12 GHz for RT5880 and 1.30–2.50 GHz, 2.91–3.12 GHz for RO4350B. The real and imaginary permeability and refractive index of the unit cell for both RT5880 and RO4350B are shown in Fig. 2c.

The real permeability of the unit cell shows positive permeability for both substrates, whereas the imaginary permeability of the unit cell displays negative permeability in the range of 2.69–2.95 GHz for RT5880 and 2.57–2.84 GHz for RO4350B. In contrast, the −ve real and imaginary frequency ranges are 2.69–2.84 GHz for RT5880, while 2.61–2.72 GHz for RO4350B substrate. However, the negative properties of the proposed MTM unit cell are the special features that might improve antenna performances. When the MTM unit cell is employed in the antenna, it enhances the bandwidth, radiation directionality, realized gain, and efficiency.

### Proposed antenna geometry and design evolution analysis

**Figure 3:**
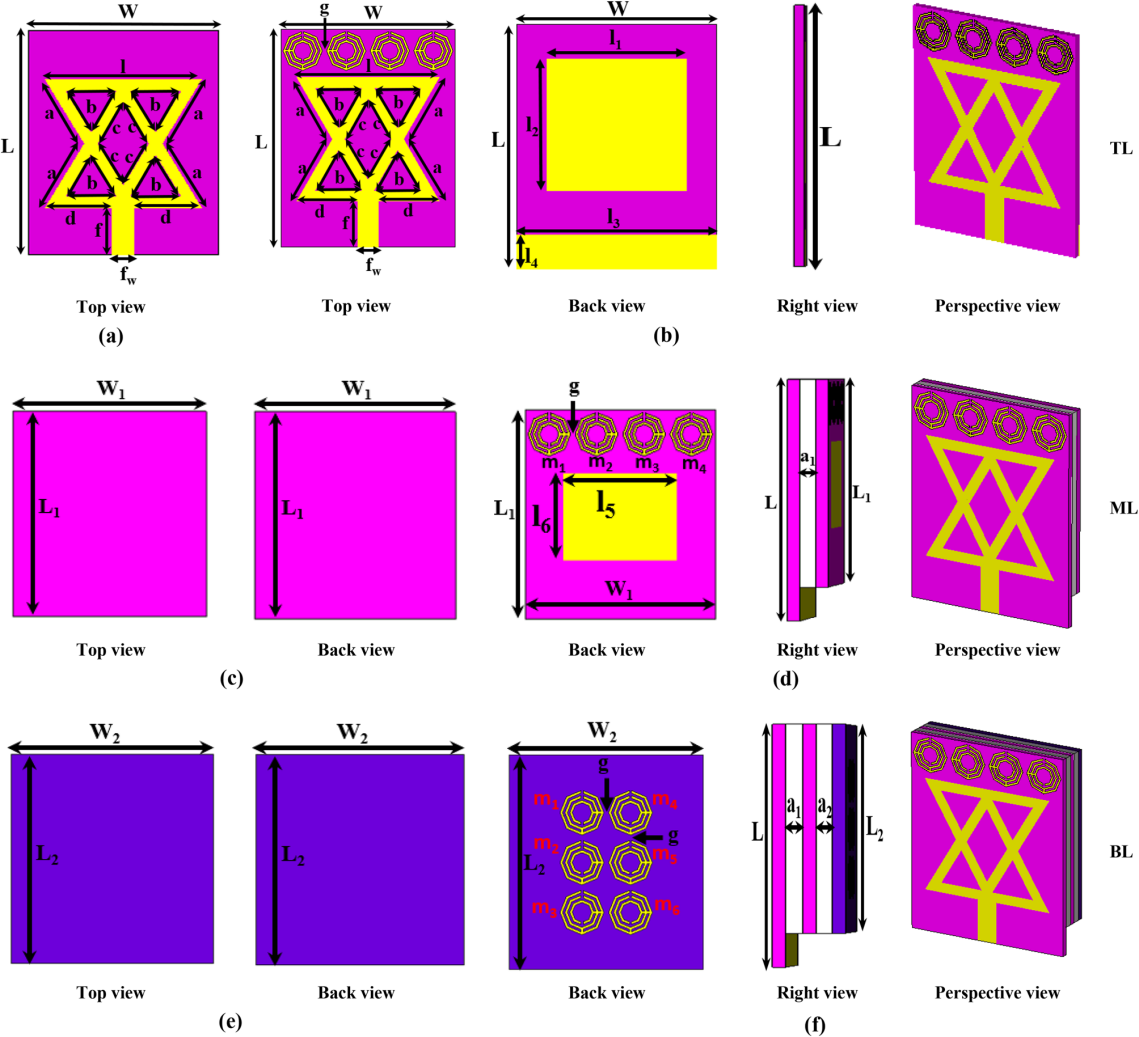
Proposed antenna geometry and evolution development: (**a**) Antenna-1(TL, without MTM); (**b**) Antenna-2 (TL, with MTM); (**c**) Antenna-3 (ML, without MTM); (**d**) Antenna-4 (ML, with MTM); (**e**) Antenna-5 (BL, without MTM); (**f**) Proposed antenna (BL, with MTM).

The wideband antenna is required in microwave brain imaging (MBI) systems with special features such as higher gain, efficiency, and directional radiation characteristics^15–18,26,37^. A wideband antenna for a microwave brain imaging system must be capable of producing a wideband operating frequency between 1 and 4 GHz with higher gain, higher efficiency, and high fidelity. If the antenna operates at wideband frequencies with the mentioned features, the signals are able to adequately penetrate to the brain tissues and the backscattered signals from the brain tissues are easily received by the receiving antennas of the system. As a result, desired high resolution-based microwave brain image generation is possible. However, frequencies below 1 GHz cause the image resolution to decrease, resulting in a noisy and indistinct image. Thus, wideband frequency is required in MBI system for detecting abnormalities (e.g., tumors) in the brain. In this article, a new MTM-loaded three-dimensional (3D) stacked wideband antenna is designed, which achieves microwave brain imaging features. The geometry and evolution of the proposed antenna are depicted in Fig. 3. The antenna consists of mainly three substrate layers: Top layer (TL), Middle layer (ML), and Bottom layer (BL), including two air gaps. The air gaps are considered between the TL and ML, ML and BL. The optimized parameters with their values are presented in Table 1. The antenna is printed on cost-effective Rogers substrate materials. The TL and ML are printed on RT5880 substrate, and the BL is printed on RO4350B substrate. In the initial step, a main radiating patch and feed line are designed on TL. Two triangle-shaped patches are attached in opposite directions to make the main radiating patch. The dimension of TL is 50 × 40 mm^2^. The partial ground (l_3_ × l_4_ mm^2^) and a rectangle-shaped parasitic element (l_1_ × l_2_ mm^2^) are attached to the backside of the TL. The lowest operating frequency (f_l_), patch width (l), and feed line width (f_w_) are calculated by the following equations^26,38,39^:

5 $$l = \frac{2c}{{3f_{r} \sqrt {\varepsilon_{r} } }}$$

6 $$f_{w} = \frac{7.48 \times h}{{e^{{\left( {z_{0} \frac{{\sqrt {\varepsilon_{r} + 1.41} }}{87}} \right)}} }} - 1.25 \times t$$

7 $$\varepsilon_{eff} = \frac{{(\varepsilon_{r} + 1)}}{2} + \frac{{(\varepsilon_{r} - 1)}}{2}\left[ {1 + \frac{10 \times h}{l}} \right]^{ - 0.5}$$

8 $$f_{l} = \frac{c}{{2 \times l\sqrt {\varepsilon_{eff} } }}$$

where *f*_*r*_ denotes the resonance frequency, *h* denotes the thickness of the substrate, *t* denotes the copper thickness, *c* denotes the speed of light in free space, *ε*_*eff*_ denotes the substrate’s effective dielectric constant. The CST simulator software is used to optimize the geometric parameters for achieving the required band and special features of the antenna. The feed line width is responsible for 50Ω impedance matching.

**Table 1:** The geometric parameters of the stacked antenna.

Parameters	Value (mm)	Parameters	Value (mm)	Parameters	Value (mm)
L	50	c	10.68	l_4_	7
W	40	d	14.05	l_5_	24
L_1_	43	f	10.50	l_6_	18
W_1_	40	f_w_	4.80	g	0.50
L_2_	43	l	32.91	a_1_	2
W_2_	40	l_1_	28	a_2_	2
a	16.45	l_2_	27	–	–
b	10.68	l_3_	40	–	–

**Figure 4:**
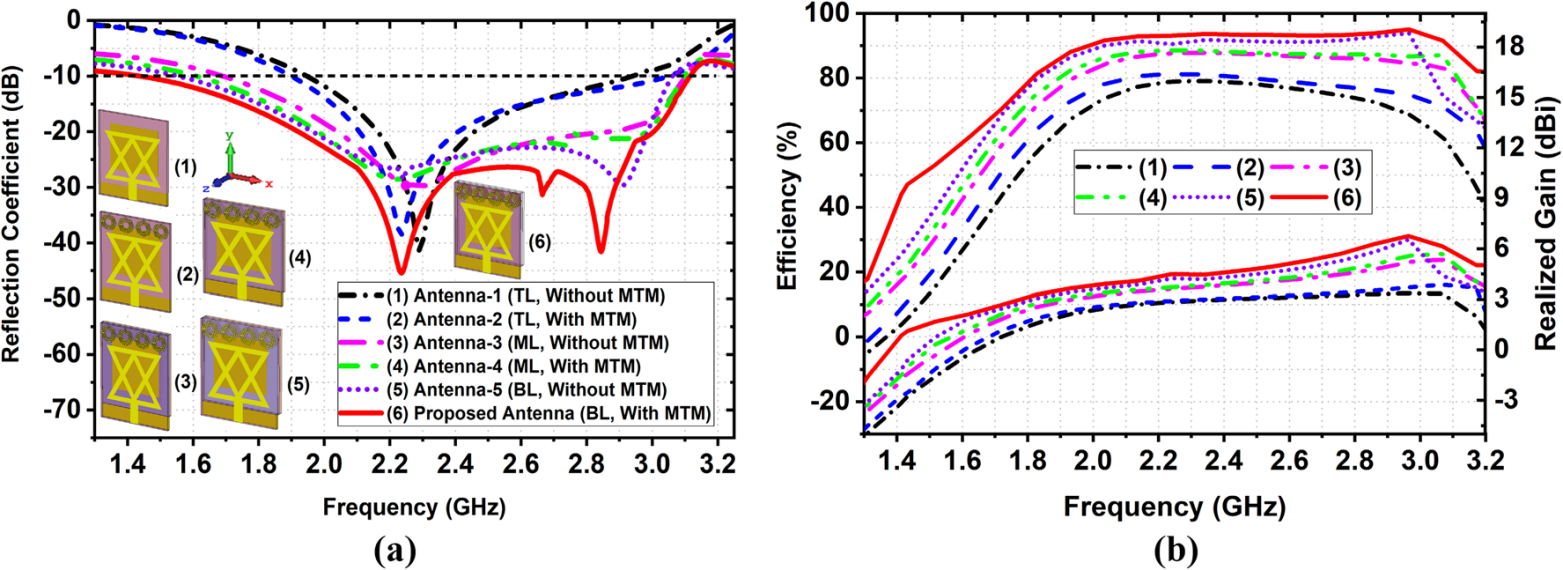
Simulated results of the different antenna design: (**a**) Reflection coefficient; (**b**) Efficiency versus Realized gain.

Figure 3 depicts the evolution of the proposed antenna. The simulated reflection coefficient |S_11_|, and efficiency vs. realized gain curves of the stacked antenna's evolution are illustrated in Fig. 4a, b, respectively. Figure 3a shows the top view of antenna-1 (TL without MTM). It is observed from Fig. 4 that the frequency band of antenna-1 is 1.94–2.94 GHz with a resonance frequency at 2.29 GHz and the maximum realized gain is 3.36dBi at 2.96 GHz with 79.13% efficiency. Thereafter, when a 1 × 4 MTM array is applied at top of the patch in antenna-2 (TL with MTM) shown in Fig. 3b, the frequency band increases towards both lower and upper frequencies due to MTM characteristics and achieves a frequency band of 1.89–3.04 GHz with a resonance at 2.24 GHz. The attained maximum realized gain of antenna-2 is 3.69 dBi at 2.96 GHz with 81.32% efficiency. In the second step, the ML (antenna-3) without MTM array is placed at 2 mm away and attached with antenna-2 by using double-sided foam tape shown in Fig. 3c. The air gap (a_1_) between antenna-2 and antenna-3 is 2 mm. The dimension of ML is 43 × 40 mm^2^ (L_1_ × W_1_). The observed operating band, gain, and efficiency of the antenna-3 are 1.69–3.10 GHz, 5.38 dBi at 2.96 GHz, and 87.84%, respectively. When another 1 × 4 MTM array (m_1_–m_4_) and a rectangular-shaped (l_5_ × l_6_) parasitic element are attached at the backside of the ML (antenna-4), as shown in Fig. 3d, the gain is increased. In addition, the radiation directivity and bandwidth are also increased. The operating frequency band is 1.61–3.10 GHz, with two resonances at 2.21 GHz and 2.92 GHz. The recorded simulated realized gain is 5.68 dBi at 2.96 GHz and 88.59% efficiency. The right view, the perspective view of the two-layered stacked antenna (antenna-4) is shown in Fig. 3d. In the third step, the BL (antenna-5) without an MTM array is positioned at 2 mm away from the ML, which is shown in Fig. 3e. The air gap (a_2_) between antenna-4 and antenna-5 is 2 mm. The dimension of BL is 43 × 40 mm^2^ (L_2_ × W_2_). The achieved frequency band, realized gain, and efficiency of antenna-5 are 1.55–3.06 GHz, 5.54 dBi, and 93.89% at 2.96 GHz, respectively. Finally, when a 3 × 2 MTM array (m_1_–m_6_) is applied at the backside of the BL (proposed antenna), the proposed antenna shows better outcomes than other antennas in terms of bandwidth, radiation directionality, realized gain, and efficiency.

The resultant simulated operating band of the proposed antenna is 1.43–3.12 GHz with a fractional bandwidth (FBW) of 74.45% for the center frequency of 2.27 GHz. It also produced three resonances at 2.24 GHz, 2.66 GHz, and 2.84 GHz. The back, right, and perspective view of the three layered-based proposed antenna is illustrated in Fig. 3f. The recorded maximum realized gain and efficiency are 6.74 dBi and 95.06% at 2.96 GHz, respectively, shown in Fig. 4b. The overall performance summary of the antenna evolutions is presented in Table 2. In the antenna design, it is noticeable that the overall performance of the antenna is enhanced due to the MTM array element effectiveness in all layers and stacked architecture. Because the negative permittivity characteristics of MTM array elements enhance the overall bandwidth. Also, it produces extra resonances and improves radiation efficiency by resulting in an additional electromagnetic coupling between radiating patches and layers. In addition, MTM unit cells increase the electrical length and enhance the current flow on the surface of the layer. As a result, it produces strong electrical coupling between layers of the antenna, which leads to improved gain, radiation directivity, and efficiency.

**Table 2:** Overall 3D stacked antenna performance.

Evaluation steps	Operating frequency (GHz)	Bandwidth (GHz)	Resonance frequency (GHz), under − 10 dB	Realized gain (dBi) at 2.96 GHz	Efficiency (%)
Antenna-1	1.94–2.94	1.00	2.29, − 41.50	3.36	79.13
Antenna-2	1.89–3.04	1.15	2.24, − 38.07	3.86	81.32
Antenna-3	1.69–3.10	1.41	2.29, − 29.91	5.38	87.84
Antenna-4	1.61–3.10	1.49	2.27, − 28.55, 2.92, − 21.21	5.68	88.59
Antenna-5	1.55–3.06	1.51	2.21, − 26.59, 2.91, − 29.47	6.54	93.89
Proposed	1.43–3.12	1.69	2.24, − 40.45, 2.66, − 31.34, 2.84, − 41.57	6.74	95.06

### Parametric analysis

**Figure 5:**
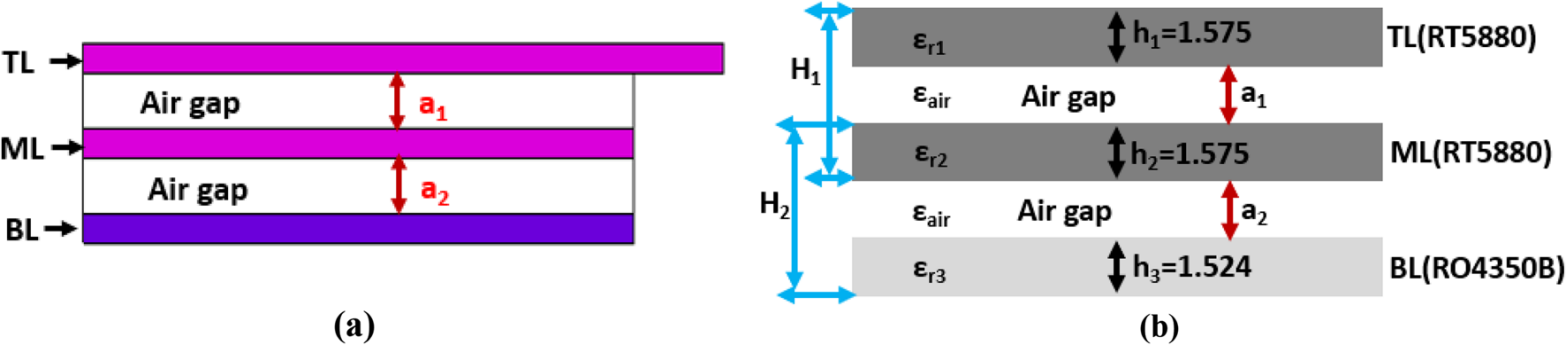
Air gaps optimization analysis: (**a**) Side view of simulated structure of the proposed antenna; (**b**) Side layout of the proposed antenna.

In Table 1, 22 parameters are presented, which are used to demonstrate the overall antenna structure. The antenna consists of three layers, and a feeding line is attached to the top layer. The ground plane is attached to the backside of the top layer. Especially, two air gaps (a_1_ and a_2_), the length of the ground plane (l_4_), and used MTM array elements in the layers are responsible for getting desired frequency band. The air gaps (a_1_ and a_2_) between layers are significant parameters to make the antenna a 3D stacked structure for better performance. The reflection coefficient is changed due to variations of air gaps between the layers. So, the air gaps must optimize to check the performance of the antenna. In simulation, we optimize the air gaps by using scientific formula. For air gaps optimization analysis, the side view simulation structure of the antenna is shown in Fig. 5a and corresponding side layout is illustrated in Fig. 5b. It is notable that the dielectric properties of the three layers (ε_r1_, ε_r2_, and ε_r3_) and air (ε_air_) are known parameters.

Also, thickness of height (h_1_, h_2_, and h_3_) of the used substrates are also known parameters, which are shown in Fig. 5b, but the conductivity of the substrates is different due to the different substrate layers properties and conductance. Thus, it effects the air gap analysis. In this case, we have to calculate the optimization value of two air gaps: a_1_ and a_2_, which are unknown parameters. According to Fig. 5b, we can calculate optimize equation for air gaps a_1_ by the following formula:

9 $$H_{1} = h_{1} + h_{2} + a_{1}$$

here H_1_ is the total distance or thickness of TL, ML, and air gap a_1_. Then, we find out the value of a_1_ from the Eq. (9) as follows:

10 $$\begin{aligned} a_{1} & = H_{1} - h_{1} - h_{2} \\ & = \left( {1.575 + 1.575 - \frac{{4\pi \sigma_{1} }}{{\omega_{1} }} - \frac{{4\pi \sigma_{2} }}{{\omega_{2} }}} \right) \\ \end{aligned}$$

where σ_1_ and σ_2_ are the conductivity of TL and ML respectively. The TL and ML also have conductive parts on the top and bottom of the substrate layers. These conductivities are same because both are same substrate. In addition, the ω_1_ and ω_2_ are the operating frequency within the frequency band 1.43–3.12 GHz and are also same because both are same substrate. However, we can choose arbitrarily any resonance frequency from this operating band. Now, arbitrarily we can calculate the optimization air gap a_1_ formula from Eq. (10) as follows:

$$a_{1} = 3.15 - 2 \times \frac{{4\pi \sigma_{1} }}{{\omega_{1} }};\quad \because \;\sigma_{1} = \sigma_{2} \;and\;\omega_{1} = \omega_{2} {\mkern 1mu}$$

for any particular frequency band. Hence, the optimization formula for the air gap a_1_ as follows:

11 $$a_{1} = 3.15 - \frac{{8\pi \sigma_{1} }}{{\omega_{1} }}.$$

Similarly, we can calculate optimization equation for air gap a_2_ by the following formula:

12 $$H_{2} = h_{2} + h_{3} + a_{2}$$

Here H_2_ is the total distance or thickness of ML, BL, and air gap a_2_. Then, we find out the value of a_2_ from the Eq. (12) as follows:

13 $$\begin{aligned} a_{2} & = H_{2} - h_{2} - h_{3} \\ & = \left( {1.575 + 1.524} \right) - \frac{{4\pi \sigma_{2} }}{{\omega_{2} }} - \frac{{4\pi \sigma_{3} }}{{\omega_{3} }} \\ \end{aligned}$$

where σ_2_ and σ_3_ are the conductivity of ML and BL respectively. The ML and BL also have conductive parts on the top and bottom of the substrate layers. These conductivities are different because both are different substrate. In addition, the ω_2_ and ω_3_ are the operating frequency within the frequency band 1.43–3.12 GHz and are also different because both are different substrate. However, we can choose arbitrarily any resonance frequency from this operating band. Now, arbitrarily we can calculate the optimization gap a_2_ formula from Eq. (13) as follows:

14 $$a_{2} = 3.1 - 4\pi \left( {\frac{{\sigma_{2} }}{{\omega_{2} }} - \frac{{\sigma_{3} }}{{\omega_{3} }}} \right).$$

**Figure 6:**
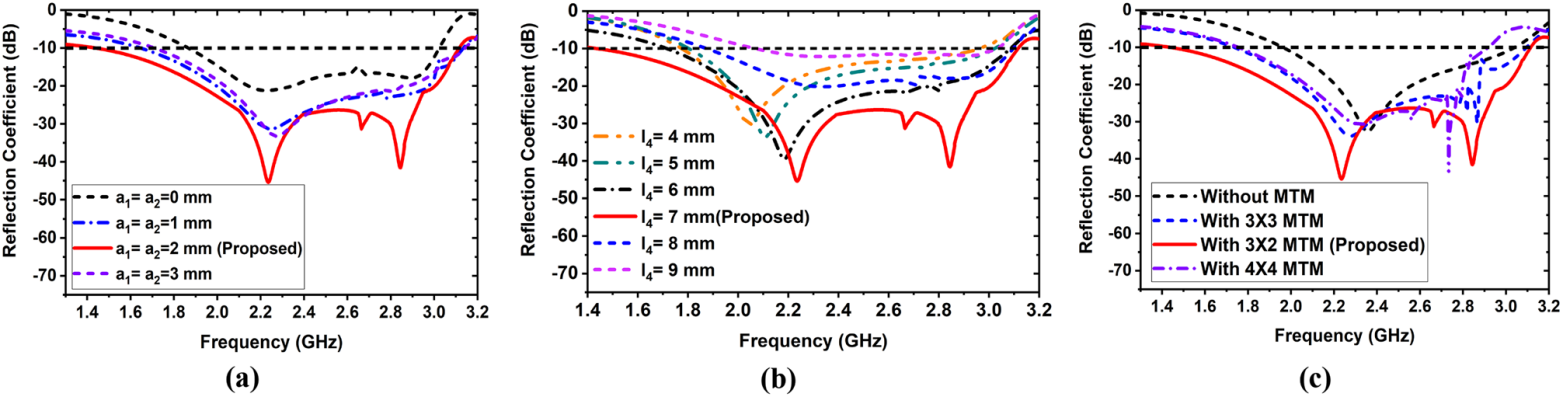
Simulated reflection coefficient of parametric analysis: (**a**) Variations of the air gaps between layers; (**b**) Variation of length of the ground plane; (**c**) Variations of the MTM array elements in the BL.

So, the Eqs. (11) and (14) are the optimization formula for air gaps a_1_ and a_2_ respectively. It is also seen that these formulas are analyzed and used during simulation. The simulated results give the optimal value as practically 2 mm for both air gaps. The simulated analysis results are illustrated in Fig. 6a. When no air gaps (a_1_ = 0 and a_2_ = 0) are considered, then the antenna shows a narrow band with a low reflection coefficient. If the middle air gap is gradually increased, the operating band is increased due to the effect of the substrate and MTM unit cell, but it is saturated at a certain value. When a_1_ = a_2_ = 1 mm, then the operating frequency band of the antenna is 1.64–3.13 GHz, with two resonances at 2.20 GHz and 2.90 GHz providing a low reflection coefficient. When a_1_ = a_2_ = 2 mm, the antenna achieved the highest operating band from the 3 mm air gap due to the air gap optimization.

The parametric analysis by considering the variations in the length of the air gaps, ground plane, and MTM unit cell are presented in Fig. 6a–c respectively. When the length of the ground plane is varied, and the remaining parameters remain constant, the reflection coefficient is demonstrated in Fig. 6b. When l_4_ = 4 mm, the achieved operating band is 1.77–2.95 GHz with a resonance at 2.05 GHz, whereas when l_4_ = 5 mm, the achieved operating band is 1.80–3.02 GHz with a resonance at 2.11 GHz. When l_4_ = 6 mm, the attained operating band is 1.71–3.08 GHz with a resonance at 2.18 GHz, whereas when l_4_ = 8 mm, the accomplished operating band is 1.87–3.09 GHz with a resonance at 2.33 GHz. If l_4_ = 9 mm is set, then the achieved operating band is 2.06–3.03 GHz with very low reflection coefficient without resonance frequency. But if the length is set l_4_ = 7 mm, then the antenna shows better outcomes than others, and the desired band is 1.43–3.12 GHz with three resonances at 2.24 GHz, 2.66 GHz, and 2.84 GHz, respectively. So, it is observed that if the ground plane length is increased the reflection coefficient is decreased.

The MTM unit cell is an important element for the antenna because the MTM array elements can change and enhance the antenna performance and increase the bandwidth, radiation directivity, and gain. In this analysis, we only showed the MTM cell effects when used in the bottom layer of the antenna. The reflection coefficient of the antenna by using the MTM array in BL layers is depicted in Fig. 6c. Initially, when no MTM unit cell is used in layers of the antenna, the antenna shows a resonance at 2.35 GHz, and the functioning band is 1.96–3.04 GHz. When 3 × 3 MTM array elements are applied to the backside of the bottom layer, the antenna's operating frequency is 1.73–3.09 GHz with two resonances at 2.27 GHz and 2.86 GHz, respectively, providing a high reflection coefficient. The operating frequency is shifted towards the lower frequencies due to MTM effects, but the resonances are distorted at the upper frequencies. The distorted resonances are also observed when 4 × 4 MTM array elements are applied and show a narrow operating band from others. But, if 3 × 2 MTM array elements are applied, then the antenna shows distorted less frequency band and it covers 1.43–3.12 GHz with three resonance at 2.24 GHz, 2.66 GHz and 2.84 GHz respectively.

## Antenna performance analysis

**Figure 7:**
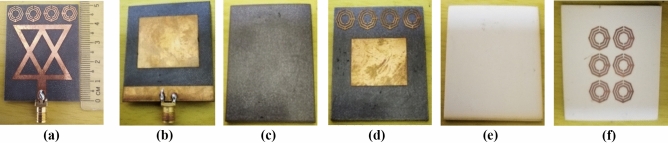
Fabricated three layers of the prototype: (**a**) Top view of TL; (**b**) Back view of TL; (**c**) Top view of ML; (**d**) Back view of ML; (**e**) Top view of BL; (**f**) Back view of BL.

**Figure 8:**
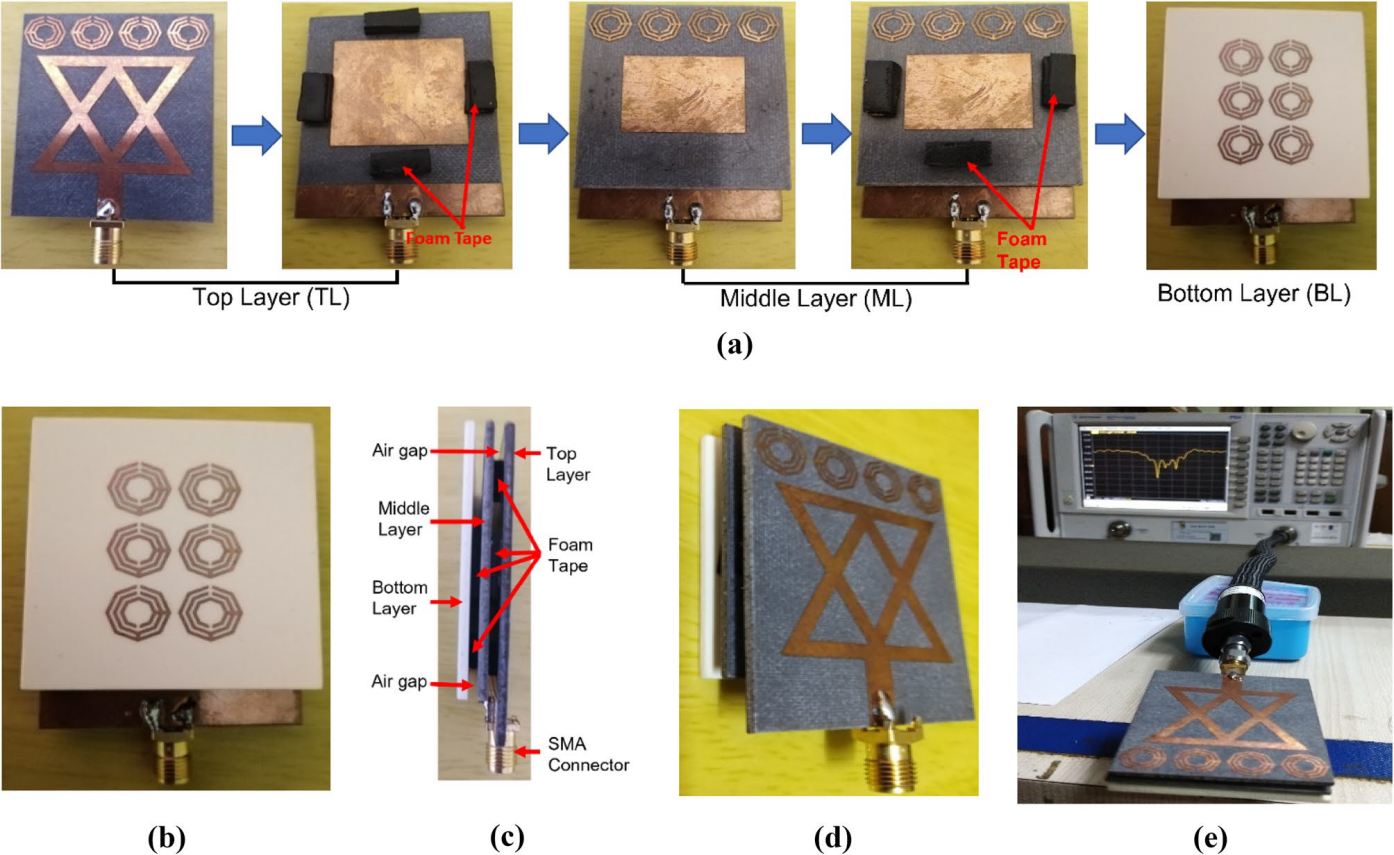
Fabricated proposed antenna and measurement: (**a**) Antenna fabrication steps; (**b**) Back view of completed antenna; (**c**) Side view of completed antenna; (**d**) Perspective view of completed antenna; (**e**) Antenna measurement by PNA.

Initially, top, middle, and bottom layers of the antenna have been printed on proposed substrate materials. Figure 7 illustrates the fabricated top view and back view of three layers of the proposed antenna. The fabricated antenna consists of three layers: Top Layers (TL), Middle Layers (ML), and Botom Layers (BL). The complete fabrication process of the proposed antenna is depicted in Fig. 8a. The ML is attached at 2 mm away from the TL using double-sided foam tape, which shown in Fig. 8a. Then, the BL has also been attached at 2 mm away from the ML by using double-sided foam tape. The thickness of the double-sided foam tape is 2 mm. Finally, the fabrication process of the antenna is completed by attaching the BL with the ML. The back view, side view, and perspective view of the fabricated antenna is illustrated in Fig. 8b–d, respectively. The fabricated antenna prototype measurement is performed by using PNA (Power Network Analyzer, PNA-N5227A). At first, the PNA is turned on and the coaxial cable is connected with the port B of PNA. After that, the frequency is set to 1–4 GHz on the PNA. Later, the PNA is calibrated by the calibration kit within the frequency range of 1–4 GHz. After that, the measurement connector is connected with the coaxial cable and then fabricated antenna is connected tightly with the connector for measurement purposes. Then, the S-parameters or reflection coefficient is observed from the PNA and recorded for further uses. The antenna measurement process is portrayed in Fig. 8e.

**Figure 9:**
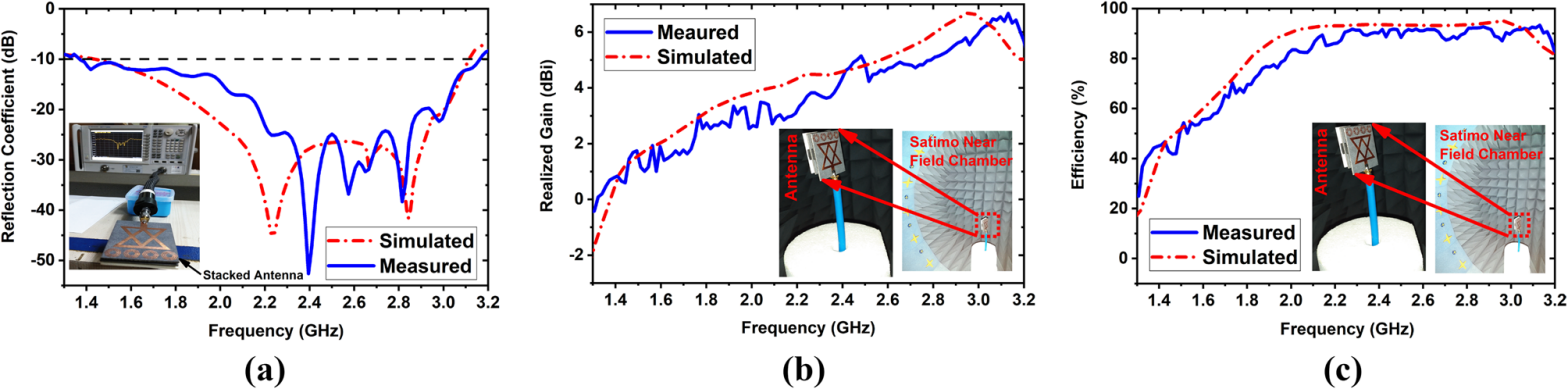
Simulated and measured outcomes of the proposed fabricated antenna: (**a**) Reflection coefficient; (**b**) Realized gain; (**c**) Efficiency.

The simulated and measured reflection coefficient is depicted in Fig. 9a. From Fig. 9a, it is observed that the simulated operating band is 1.43–3.12 GHz with 74.45% fractional bandwidth (FBW), whereas the measured operating band is 1.37–3.16 GHz with 79.20% FBW. It is seen in the measured result that the antenna generates three resonances at 2.39 GHz, 2.57 GHz, and 2.81 GHz under − 35 dB. The first resonance has shifted towards the upper frequency from 2.24 to 2.39 GHz (150 MHz). The second resonance has shifted towards a lower frequency from 2.66 to 2.57 GHz (90 MHz) third resonance is approximately the same as the simulated resonance. In Fig. 9a, it is seen that the there is a difference between simulated and measured results. This scenario may be happened due to the fabrication tolerance, calibration inaccuracy, and mutual coupling effect of layers. It is also seen that the measured reflection peak at first resonance is dipper compared to the simulated results. This minor discrepancy exists due to some factors: (i) changing loss tangent of substrate materials when measured in free environment, (ii) stacked structure of the antenna when the layers are attached using foam tape, (iii) mutual coupling effect between layers and MTM effectiveness. The loss tangent of substrate can be changed due to the change of temperature, and mutual coupling effect of MTM array always produce minor variations. As a result, it is shown difference between simulated and measured results and produce dipper reflection peak. However, the measured and simulated outcomes demonstrated good agreement between them. In addition, the Satimo near filed chamber has been used to perform the measurement of the radiation pattern, realized gain, and efficiency of the fabricated prototype. Figure 9b,c illustrate the antenna’s measured and simulated realized gain and efficiency, respectively. It is observed from Fig. 9b that the measured maximum realized gain is 6.67 GHz at 3.13 GHz, whereas the simulated maximum realized gain is 6.74 GHz at 2.96 GHz. Also, Fig. 9c investigated the maximum measured efficiency of 94%, whereas the simulated maximum efficiency is 95.06%. The simulated and measured outcomes exhibited good agreement between them in both gain and efficiency.

**Figure 10:**
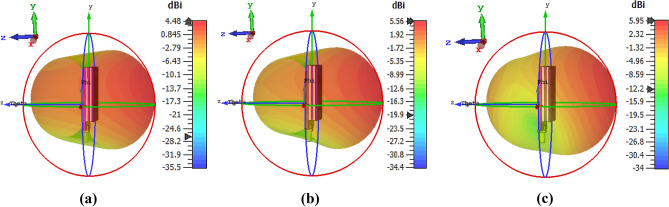
3D radiation pattern of the proposed antenna at: (**a**) 2.24 GHz; (**b**) 2.66 GHz; (**c**) 2.84 GHz.

**Figure 11:**
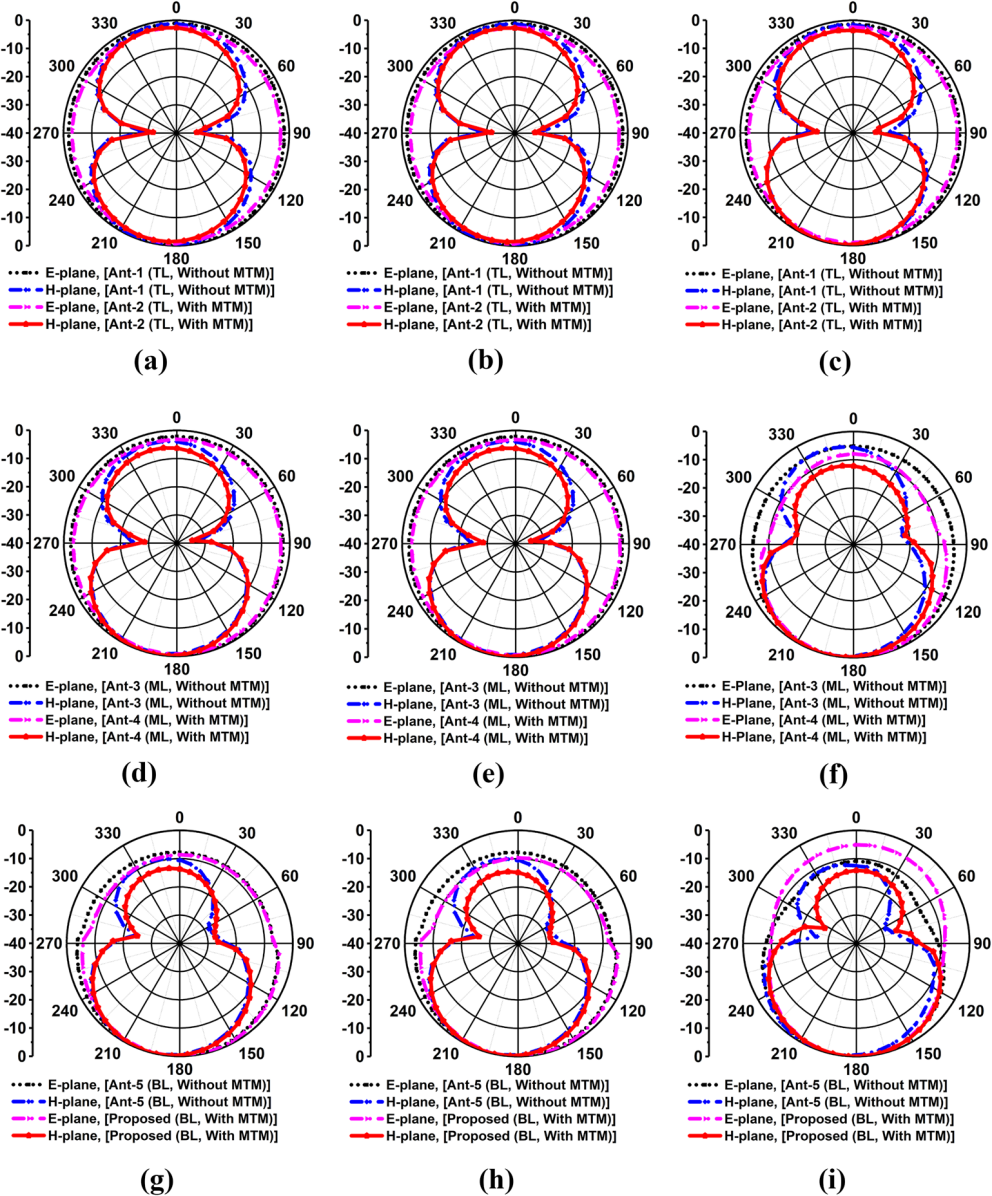
Simulated 2D radiation pattern (Far-field) in E-plane and H-plane of the antenna’s evolution for: (**a**) Ant-1 and Ant-2 at 2.24 GHz; (**b**) Ant-1 and Ant-2 at 2.66 GHz; (**c**) Ant-1 and Ant-2 at 2.84 GHz; (**d**) Ant-3 and Ant-4 at 2.24 GHz; (**e**) Ant-3 and Ant-4 at 2.66 GHz; (**f**) Ant-3 and Ant-4 at 2.84 GHz; (**g**) Ant-5 and proposed at 2.24 GHz; (**h**) Ant-5 and proposed at 2.66 GHz; (**i**) Ant-5 and proposed at 2.84 GHz.

It is noticeable that the directional radiation characteristic of the antenna is required in microwave brain imaging applications. Thus, the radiation patterns of the proposed antenna are investigated. Figure 10 illustrates a 3D radiation pattern of the antenna. It is seen that the antenna shows directional radiation characteristics, which satisfies the imaging system requirements. It is also notable that the antenna must operate in the far-field region when it works in the brain imaging system. Thus, the far-field radiation investigation is necessary because of the high permittivity of the human brain. The simulated 2D far-field radiation patterns in the E-plane (φ = 0°) and H-plane (φ = 90°) of layer-wise antenna evolution (Ant.-1, Ant.-2, Ant.-3, Ant.-4, Ant.-5 and proposed) at 2.24 GHz, 2.66 GHz, and 2.84 GHz are illustrated in Fig. 11a–i. In this research, designed a metamaterial loaded three-dimensional wideband stacked antenna for detecting brain tumors. The wideband antenna is required in microwave brain imaging (MBI) systems with special features such as higher gain, efficiency, and directional radiation characteristics. Designing a wideband antenna with directional radiation and gain is challenging for brain imaging due to frequency band limit within the range of 1–4 GHz. Previously, several staked wideband antennas have been developed for microwave communications and many other antennas have been developed for microwave imaging systems, but these are failed to generate high resolution image due to lack of low gain and radiation directivity. According to best of authors knowledge, this is the first new metamaterial based stacked antenna which is applied in microwave brain imaging system to identify the target tumors in the brain. The proposed stacked antenna achieved high gain with directional radiation characteristics within the operating band of 1.37–3.16 GHz. Also, the antenna achieved high fidelity factor (FF) of 98% that ensures the very low distortion, and the signal can sufficiently penetrate to the head tissue. The MTM unit cell is utilized in three layers to ensure the directional radiation of the antenna. The MTM based proposed antenna led to a drastic improvement in the antenna’s directional radiation properties. For better explanation, the effectiveness of MTM for improving radiation directionality of the antenna is shown in Fig. 11. It is noticeable that the directional radiation characteristic of the antenna is required in microwave brain imaging applications. It is observed from Fig. 11 the back lobe is reduced due to using the MTM on the stacked layers of the antenna, as well as no side loop is created. As a result, the front-to-back ratio (FBR), gain, radiation efficiency, and directivity of the antenna are increased. In addition, the radiation directivity at a higher frequency is more directive rather than lower frequency. So, obviously it is inferred that the proposed stacked antenna has led to a drastic improvement in the antenna radiation properties. Also, the radiation pattern in the H-plane of the antenna exhibits successively wider beamwidth towards the boresight direction than E-plane. That means the antenna mostly radiated towards the  − Z direction.

**Figure 12:**
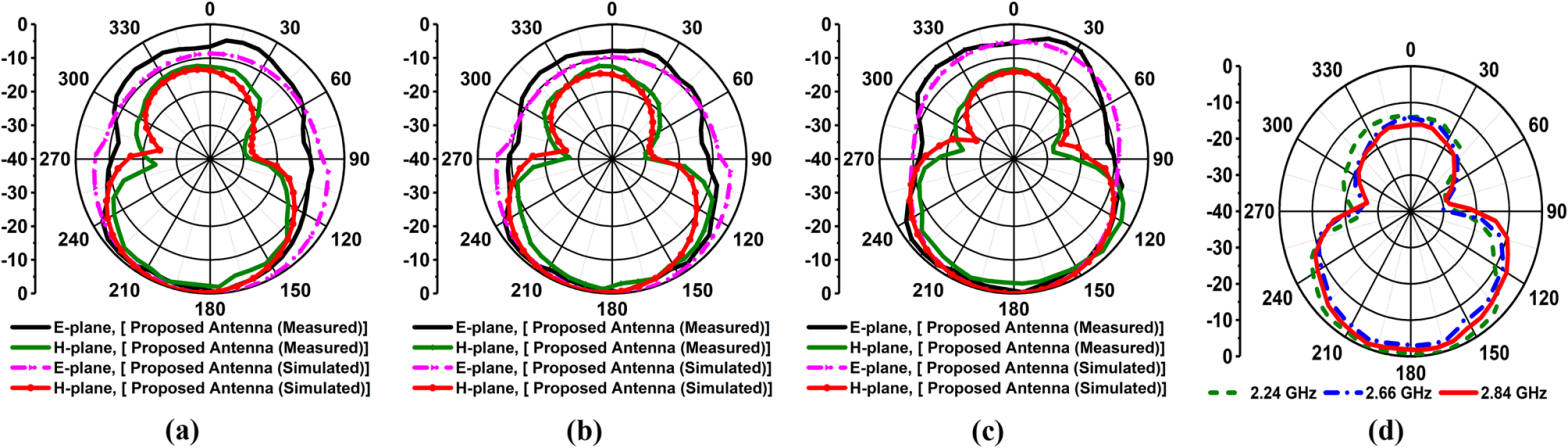
Simulated and measured radiation pattern in E-plane and H-plane of the proposed antenna at: (**a**) 2.24 GHz; (**b**) 2.66 GHz; (**c**) 2.84 GHz; (**d**) Measured near-field in H-plane at 2.24 GHz, 2.66 GHz, and 2.84 GHz.

The simulated and measured far-field radiation pattern at 2.24 GHz is shown in Fig. 12a. Figure 12b illustrates the simulated and measured far-field radiation pattern at 2.66 GHz, and simulated and measured far-field radiation pattern at 2.84 GHz is shown in Fig. 12c. The measured and simulated radiation patterns showed good agreement between them in the entire operating band. Moreover, the antenna will be placed near the head model for imaging purposes; hence it is needed to verify the near field characteristics of the antenna. Thus, the antenna is measured at the near field lab. Figure 12d presents the near-field radiation measurement in the H-plane scenario. It is examined proposed prototype demonstrates a directional characteristic for 2.24 GHz, 2.66 GHz, and 2.84 GHz, respectively, and shows almost symmetrical for both far-field and near-field. Furthermore, the fidelity factor (FF) is calculated to investigate the antenna near field performances by applying two methods: (i) using E- field probs, and (ii) using transmitting and receiving antenna by considering the near-field distance between them in four scenarios. Typically, the FF is used to evaluate the level of distortion in the near-field region, which is defined by the correlation coefficient between the radiated received E-field pulses *E*_*rad*_ in various directions (φ, θ) and the exciting Gaussian pulses *G*_*t*_ at the input of the antenna. In this work, the finite difference time domain (FDTD) method is utilized to investigate the level of distortion in both methods. Firstly, to observe the FF and time-domain amplitude responses around the antenna, E-field probes are situated around the antenna at distances of 50 mm from the center of the antenna and arranged in both the E-plane and H-plane with angular differences of 15° apart from each other. The FF at a specific direction is calculated as^14,26^:

15 $$FF(\varphi ,\theta ) = \max \frac{{\int\limits_{ - \infty }^{ + \infty } {E_{rad} (t,\varphi ,\theta ).G_{t} (t - \tau ,\varphi ,\theta )dt} }}{{\sqrt {\int\limits_{ - \infty }^{ + \infty } {\left| {E_{rad} (t,\varphi ,\theta )} \right|^{2} dt\int\limits_{ - \infty }^{ + \infty } {\left| {G_{t} (t,\varphi ,\theta )} \right|^{2} dt} } } }}.$$

**Figure 13:**
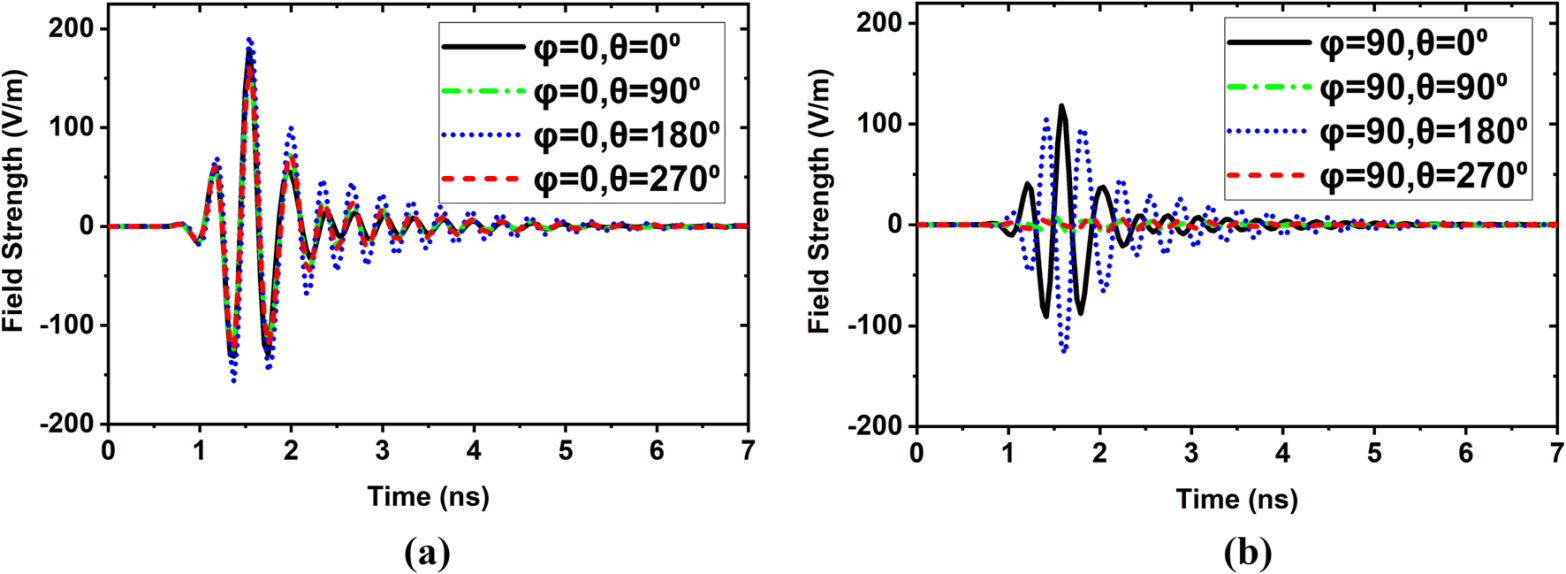
Near field measurements in time domain at different angles: (**a**) Received E-filed radiated pulse in E-plane; (**b**) Received E-filed radiated pulse in H-plane.

**Figure 14:**
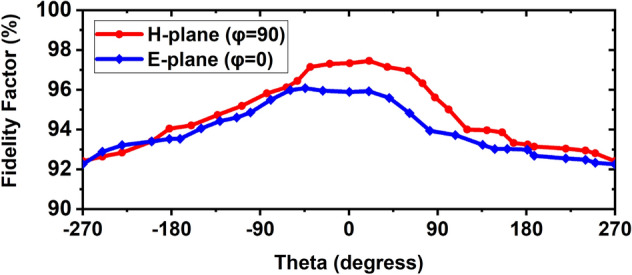
Fidelity factor (FF) measurements in E-plane and H-planes.

**Figure 15:**
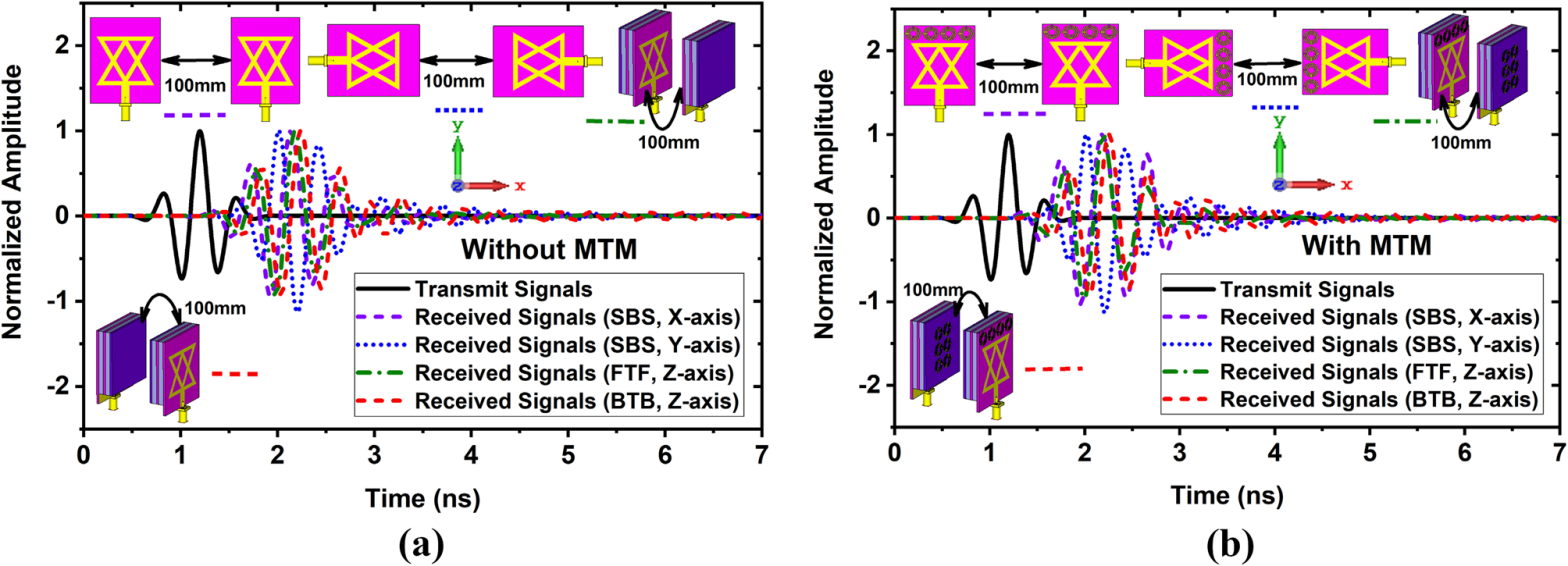
Normalized received signals in four orientations: (**a**) Without MTM; (**b**) With MTM.

Figure 13a,b depicts the radiated received E-field pulses in Near field regions at different angles. It shows the antenna radiates almost distortionless pulses. The calculated fidelity factors for E- and H-plane are illustrated in Fig. 14. It is seen from Fig. 14 that the FF is greater than 93% in both E and H-planes, and the maximum FF is 98.01% for H-plane and 96% for E-plane. Secondly, since the antenna will be utilized in the imaging system to receive scattered signals, where the distance of antenna to antenna is 100 mm, thus, it is needed to investigate the fidelity factor of the antenna at a different axis. In this work, four cases are considered: (i) Side by side at X-axis (SBS, X-axis), (ii) Side by side at Y-axis (SBS-Y-axis, (iii) Front to Front at Z-axis (FTF, Z-axis), and (iv) Back-to-back at Z-axis (BTB, Z-axis). The normalized transmitting and receiving signals for four cases are illustrated in Fig. 15a,b. It is examined that when an MTM unit cell is applied to the antenna in different layers, the antenna receives almost distortionless pulses than the without an MTM-based antenna. As a result, the proposed antenna (MTM-based) exhibits a higher fidelity factor for four cases. The calculated FFs are 84.42%, 86.24%, 90.93%, and 91.01% for SBS-X-axis, SBS-Y-axis, FTF-Z-axis, BTB-Z-axis without MTM respectively, whereas 93.41%, 94.52%, 95.20% and 98% for SBS-X-axis, SBS-Y-axis, FTF-Z-axis, BTB-Z-axis with MTM respectively. However, it is concluded that the proposed antenna is performed better in both far-field and near-field. Thus, it is a suitable candidate in the microwave brain imaging system, where the antenna has to be set up in a Back-to-Back (BTB, Z-axis) orientation to get better performance.

## Sensitivity measurements of the head model

**Figure 16:**
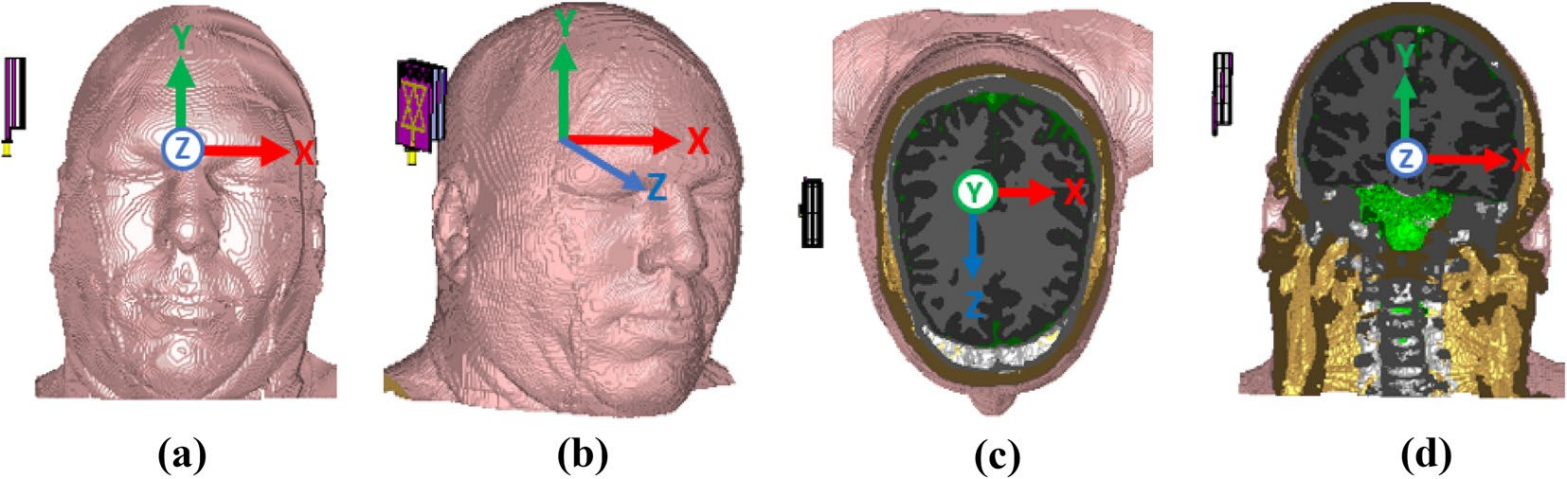
Different views and planes of Hugo Head model with a single antenna: (**a**) Front view; (**b**) Perspective view; (**c**) Transverse plane; (**d**) Coronal Plane.

**Figure 17:**
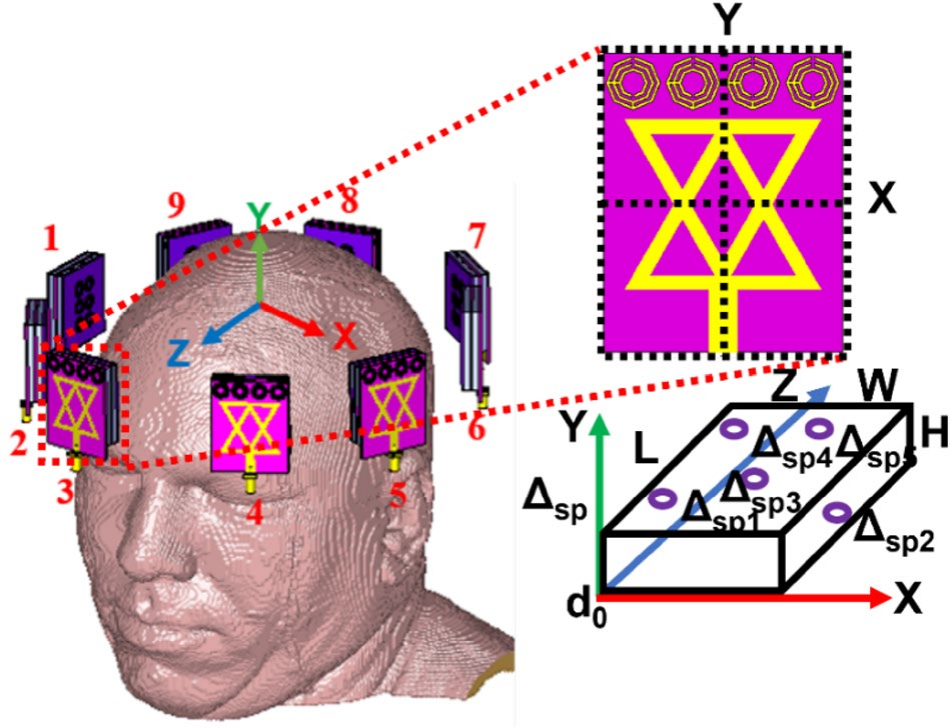
Arbitrary current and voltage picking point of stacked antenna and impedance matching scenario between antenna and Hugo model.

In this section, we discuss the sensitivity analysis, which refers to investigating the antenna's scattering parameters (S-parameters) with a benign and a malignant tumor as brain abnormalities in the Hugo head model, signal penetration, and specific absorption rate (SAR). The Hugo head model is imported from the CST 2019 software’s voxel model. Different views and cutting plane views of the Hugo model are depicted in Fig. 16. It is notable that the impedance matching between antenna and Hugo head model is necessary for better imaging outcomes. So, the impedance matching is also investigated during simulation analysis. The stacked structure of the antenna is a combination of coupling and decoupling of patches and MTM elements with air gap between layers as a dielectric medium. Consequently, at any moment, in order to calculate the impedance between the transmitting antenna and the Hugo head model, the transmitting antenna with excitation at any point of the surrounding imaging setup can be analytically optimized. The Iteratively Corrected Coherence Factor Delay-Multiply-and-Sum (IC-CF-DMAS) imaging algorithm^17^ is used for impedance optimization by utilizing the Green’s function^40^. For instance, the real Green’s function does not reflect the insignificant surface current and voltage difference at an arbitrary point of any microstrip line^40^. The rectangular shape is judged for the antenna demonstrated in Fig. 17 assuming instantaneous transmitting antenna component and Hugo model for matching the impedance.

When defining any random surface point (∆sp) in the microstrip coupled line (MCL), the antenna's 3D structure plane is expected to be the same as in simulations, along with the antenna's length (L), width (W), and height (H). The effective permittivity (ε_eff_), substrate permittivity (ε_sp_), air permittivity (ε_air_), and loss tangent (tanδ) considered between Hugo model and the antenna. So, random surface points (∆sp1 to ∆sp5) are decided on the MCL as the assessment location of current and voltage estimation for impedance matching. Rewrite the function as follows to distinguish it from the original Green's function and include precise current and voltage at a random MCL surface location in the simulated imaging setup with antenna and Hugo model:

16 $$Gn_{obs(1 \ldots 5)} = \frac{1}{LW}\sum\limits_{m = 0}^{\infty } {\sum\limits_{n = 0}^{\infty } {(2 - \delta_{m} )(2 - \delta_{n} ) \times \frac{{\varepsilon_{eff} \varepsilon_{air} \varepsilon_{sp} Cos(P_{x} x)Cos(P_{y} y)}}{{P_{mn}^{2} - P^{2} }}} }$$

where $P_{mn}^{2} = P_{x}^{2} + P_{y}^{2}$, $P_{x} = (m\pi /L)$, $P_{y} = (n\pi /W)$, and m, n = 0,1,2 … ∞ is the propagation mode deemed on the rapid transmission. The permittivity (µ) and antenna height (H) associated to higher-order frequency terms have been removed from the real functions because their convergence mechanisms slow down the calculation as a whole. The imaging algorithm now incorporates Eq. (16) and translates or postulates it as a different solution that uses coupling and decoupling lines for impedance matching. However, this technique works well when there are multiple radiating antennae or boundaries surrounding the Hugo model. If the boundary is ignored, the contribution from the excitation port in the electromagnetic field that reaches the observation point can be assumed, and effective dielectric parameters speed up the calculation accuracy of the provided imaging system for impedance matching.

**Figure 18:**
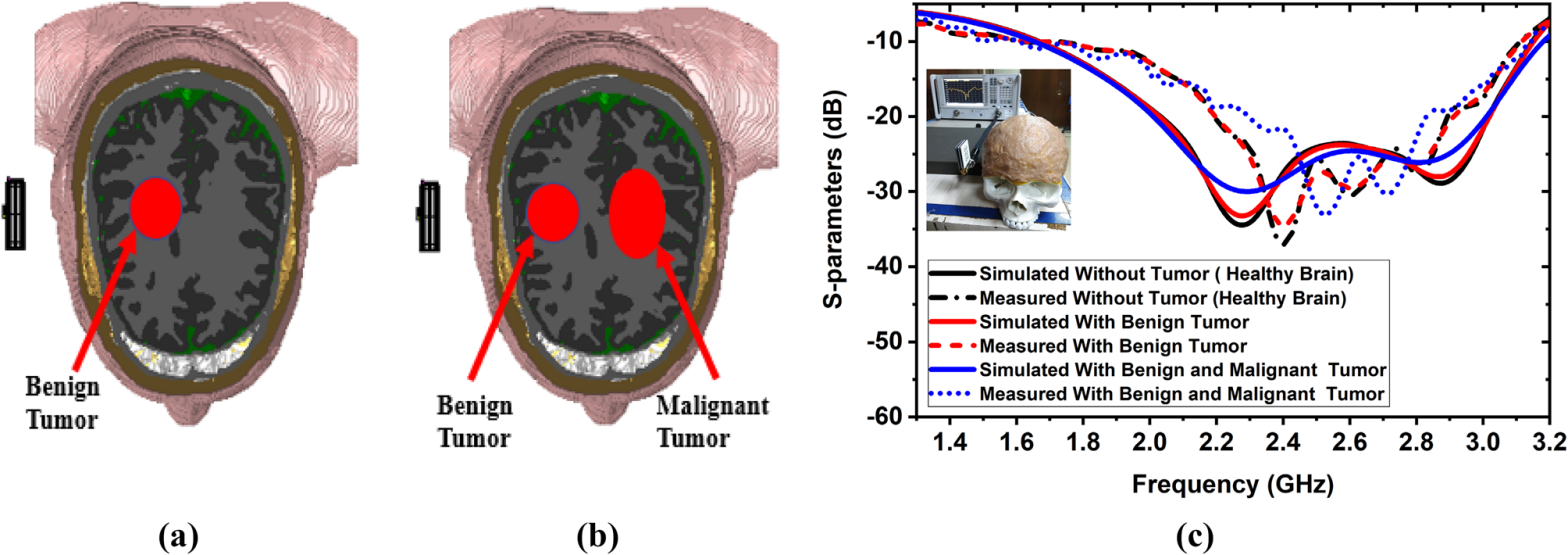
Simulated and measured results with head model including tumors: (**a**) Head model with a benign tumor; (**b**) Head model with a benign and a malignant tumor; (**c**) S-parameters.

**Figure 19:**
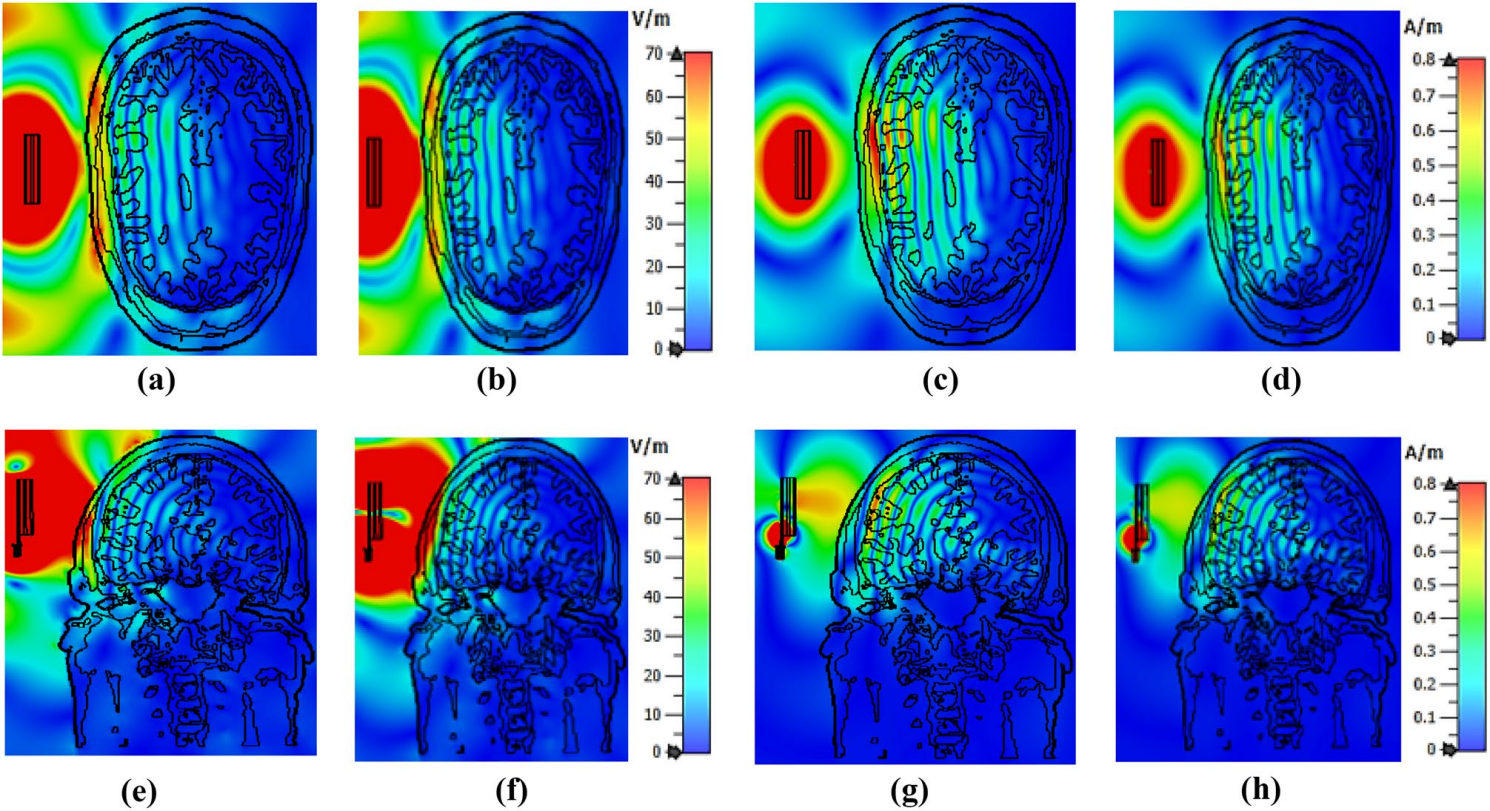
Microwave signal penetration distribution to the head model: (**a**) E-field distribution in xz plane at 2.24 GHz; (**b**) E-field distribution in xz plane at 2.84 GHz; (**c**) H-field distribution in xz plane at 2.24 GHz; (**d**) H-field distribution in xz plane at 2.84 GHz; (**e**) E-field distribution in yz plane at 2.24 GHz; (**f**) E-field distribution in yz plane at 2.84 GHz; (**g**) H-field distribution in yz plane at 2.24 GHz; (**h**) H-field distribution in yz plane at 2.84 GHz.

A six-layered tissue-mimicking head phantom model with different layers and tumor(s) is fabricated to investigate the antenna's performance (described in “Microwave brain imaging results and discussion” section). The tumors are placed at different positions in the model. Figure 18a represents the head model with a single benign tumor, and Fig. 18b represents the head model with a benign and a malignant tumor. The simulated and measured (details are discussed in “Microwave brain imaging results and discussion” section) S-parameters of the head model with and without tumor using a single antenna are presented in Fig. 18c. The reflection coefficients are slightly decreased with resonance frequencies and shifted towards higher frequency. It is observed from Fig. 18c, there is slight difference between simulated and measured outcomes. This scenario can be occurred due to the change of head tissues and tumor dielectric properties. In this research, we have simulated a realistic Hugo head model, including tumors, where the thickness and dielectric properties of the tissues are fixed. On the other hand, the fabricated tissues thickness and dielectric properties are slightly changed due to fabrication tolerance (i.e., thickness and combination of ingredients). Also, the free space measurement, environmental temperature, time variant factors are affected the measured result. As a result, there is a difference between simulated and measured results, but remain stable within the required operating band because of the effectiveness of the MTM array. However, the antenna showed a good agreement between simulated and measured outcomes. In this work, the microwave signal penetration to the head is also investigated to evaluate antenna effectiveness. Figure 19 illustrates the E-and H-filed penetration inside the head model. The E-field and H-field distribution in the xz-plane at 2.24 GHz and 2.84 GHz are shown in Fig. 19a–d, respectively. Besides, The E-field and H-field distribution in the yz-plane at 2.24 GHz and 2.84 GHz are shown in Fig. 19e–h, respectively. It is observed from Fig. 19; that the antenna continues to show the directionality to the model, and the signal is able to penetrate the head tissues covering a two-thirds portion of the head.

**Figure 20:**
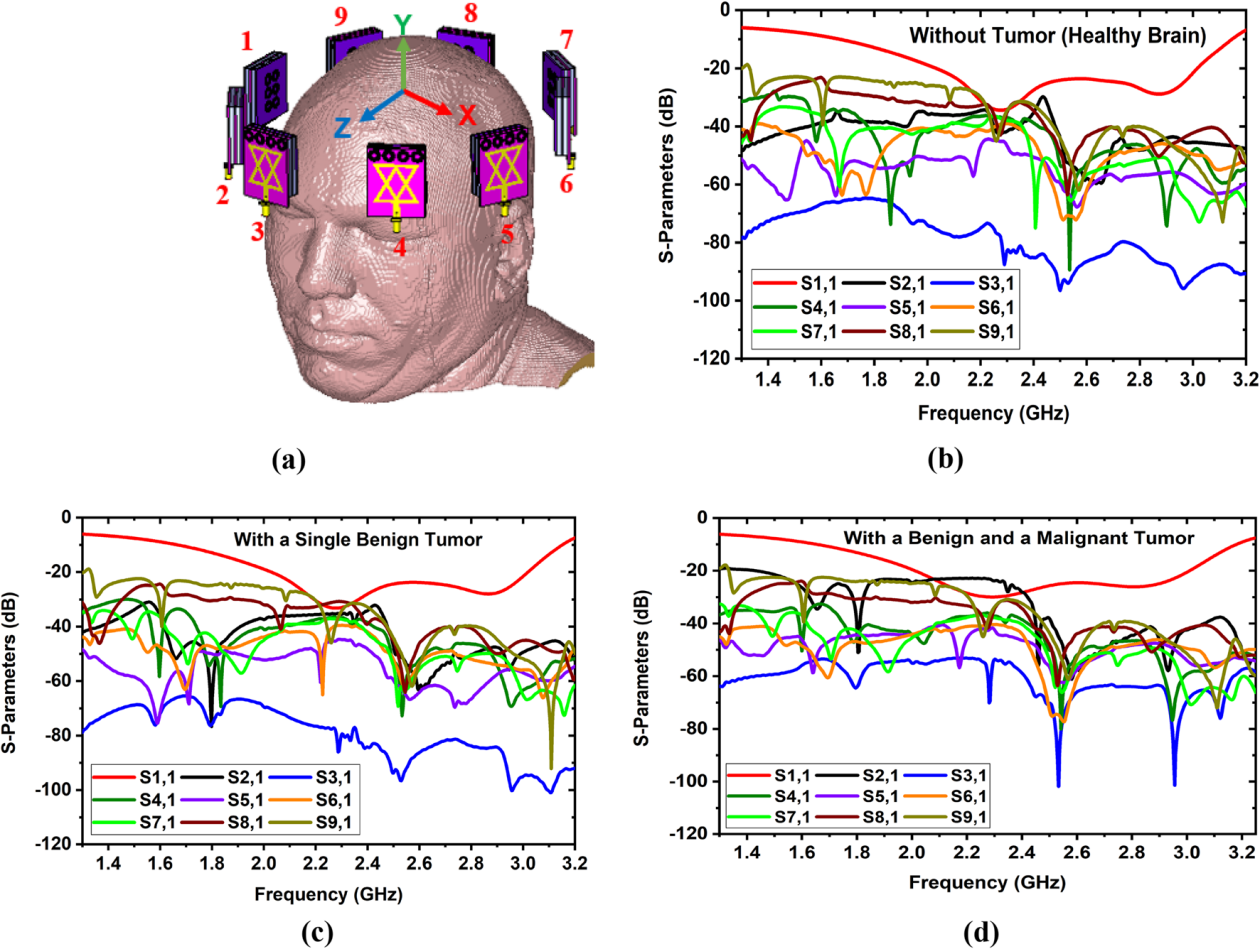
Simulated imaging setup and measurements: (**a**) Nine-antenna array setup with head; (**b**) S-parameters without tumor; (**c**) S-parameters with a single benign tumor; (**d**) S-parameters with a benign and a malignant tumor.

Figure 20a illustrates the simulated nine-antenna array set up, where antenna 1 acts as a transmitter and the remaining eight antennas act as receivers. The received S-parameters without tumor (i.e., Healthy brain), with a single benign tumor, and a single benign and a malignant tumor are depicted in Fig. 20b,d, respectively. It is examined from the S-parameters that there is significant distortion of the backscattered signals for the benign and malignant tumors. These distortion differences happened due to the presence of high dielectric properties of the tumors and peak resonance frequencies algorithm. However, the nine-antenna array setup covers the complete area of the head, which carries all the sufficient information for reconstructing the brain images. Also, the specific absorption rate (SAR) is analysed because microwave radiation is extremely harmful to the human brain when exposed to the brain. So, the SAR analysis is an essential consideration for microwave brain imaging modalities to ensure operational safety. The SAR is measured by the following formula^41^:

17 $$SAR = \frac{{\left| {E_{f} } \right|^{2} .\sigma }}{{M_{d} }}$$

where $E_{f}$ represents the electric fields, $M_{d}$ represents the mass density, and $\sigma$ represents the conductivity of the human brain tissue. According to the IEEE radiation exposure standard regulations, the highest SAR must not exceed 1.6 W/kg for 1 gm and 2 W/kg for 10 gm of tissue^41,42^. In this research, 1mW power is applied as an input to the antenna (positions: 1, 3,5,7, and 8) and observed the SAR at 2.24 GHz, 2.66 GHz, and 2.84 GHz, respectively, for 1 gm and 10 gm of tissue. The measured SAR values are presented in Table 3. The investigation shows that the observed maximum SAR value is 0.0020 W/Kg for 1gm and 0.0018 W/kg for 10 gm of tissue with the proposed MTM loaded 3D stacked antenna at 2.66 GHz, which satisfies the IEEE radiation exposure limit. Also, it is realized from Table 3, that when MTM array elements are applied to the antenna, the SAR value is decreased. The observed highest SAR value is 0.0047 W/kg and 0.0029 W/kg for 1 gm and 10 gm of tissue without MTM array components, respectively, whereas the value is 0.0020 W/kg and 0.0018 W/kg for 1 gm and 10 gm of tissue for the proposed stacked antenna at 2.66 GHz. Therefore, it is concluded that the proposed staked antenna is applicable in microwave brain imaging systems to reduce SAR. In this research, it is noticeable that the SAR value is observed from different side of the head model by placing the antenna at different positions. It is also observed from Table 3, higher SAR value is recorded for higher gain of the antenna whereas lower SAR value is recorded for lower gain of the antenna. This circumstance is occurred due to the absorbed microwave energy by the head tissues and effectiveness of dielectric properties (i.e., permittivity and conductivity) of brain tissues. The head model is a multilayer structure consists of six layers. The permittivity of brain tissues is decreased with respect to increasing the frequency, while conductivity is increased with respect to increasing frequency. If the permittivity and conductivity is varied with the variations of frequency and gain of the antenna, the backscattered signals are also reflected and scattered at different manner. Therefore, when the gain of the antenna is low at lower frequencies, then the radiation directivity is partial directional. So, the microwave signal cannot fully penetrate to the brain tissues and the absorbed energy by the tissues is also low. Hence, the calculated SAR value is low. On the other hand, when the gain gradually increased at higher frequencies, then the radiation directivity is fully directional which is controlled by MTM array. Consequently, the radiated microwave signal can sufficiently penetrate to the brain tissues and higher energy absorbed by the brain tissues. As a result, the calculated SAR value is high, but it is safe for human brain and not exceed IEEE standard safety limit.

**Table 3:** Performance assessment of the antenna with and without MTM elements for SAR at different frequencies.

Antenna position (1,3,5,7,8)	Freq. (GHz)	Maximum SAR (W/Kg)											
		With MTM						Without MTM					
		1 gm			10 gm			1 gm			10 gm		
		TL	ML	Proposed	TL	ML	Proposed	TL	ML	BL	TL	ML	BL
	2.24	0.0026	0.0020	0.0016	0.0021	0.0018	0.0013	0.0053	0.0046	0.0036	0.0033	0.0027	0.0023
	2.66	0.0028	0.0021	0.0018	0.0023	0.0017	0.0013	0.0047	0.0044	0.0040	0.0035	0.0031	0.0025
	2.84	0.0029	0.0022	0.0019	0.0025	0.0016	0.0012	0.0046	0.0034	0.0038	0.0032	0.0026	0.0021
	2.24	0.0022	0.0020	0.0012	0.0018	0.0010	0.0008	0.0040	0.0035	0.0025	0.0031	0.0027	0.0022
	2.66	0.0021	0.0019	0.0011	0.0009	0.0008	0.0007	0.0038	0.0030	0.0024	0.0029	0.0023	0.0020
	2.84	0.0020	0.0014	0.0011	0.0009	0.0007	0.0005	0.0037	0.0031	0.0023	0.0025	0.0020	0.0019
	2.24	0.0039	0.0032	0.0029	0.0027	0.0022	0.0017	0.0050	0.0045	0.0043	0.0038	0.0032	0.0026
	2.66	0.0040	0.0033	0.0020	0.0028	0.0024	0.0018	0.0054	0.0050	0.0047	0.0039	0.0033	0.0029
	2.84	0.0021	0.0017	0.0010	0.0009	0.0006	0.0005	0.0053	0.0046	0.0041	0.0038	0.0030	0.0026
	2.24	0.0022	0.0018	0.0012	0.0009	0.0007	0.0006	0.0048	0.0040	0.0030	0.0038	0.0028	0.0018
	2.66	0.0021	0.0016	0.0010	0.0008	0.0005	0.0004	0.0035	0.0030	0.0025	0.0025	0.0020	0.0015
	2.84	0.0020	0.0015	0.0011	0.0008	0.0005	0.0004	0.0034	0.0029	0.0025	0.0024	0.0018	0.0015
	2.24	0.0028	0.0022	0.0018	0.0021	0.0015	0.0012	0.0039	0.0034	0.0029	0.0034	0.0024	0.0014
	2.66	0.0027	0.0023	0.0017	0.0021	0.0015	0.0011	0.0038	0.0033	0.0030	0.0035	0.0025	0.0015
	2.84	0.0025	0.0018	0.0015	0.0010	0.0009	0.0008	0.0052	0.0048	0.0046	0.0037	0.0029	0.0022

## Microwave brain imaging results and discussion

### Phantom fabrication process and measurement

**Figure 21:**
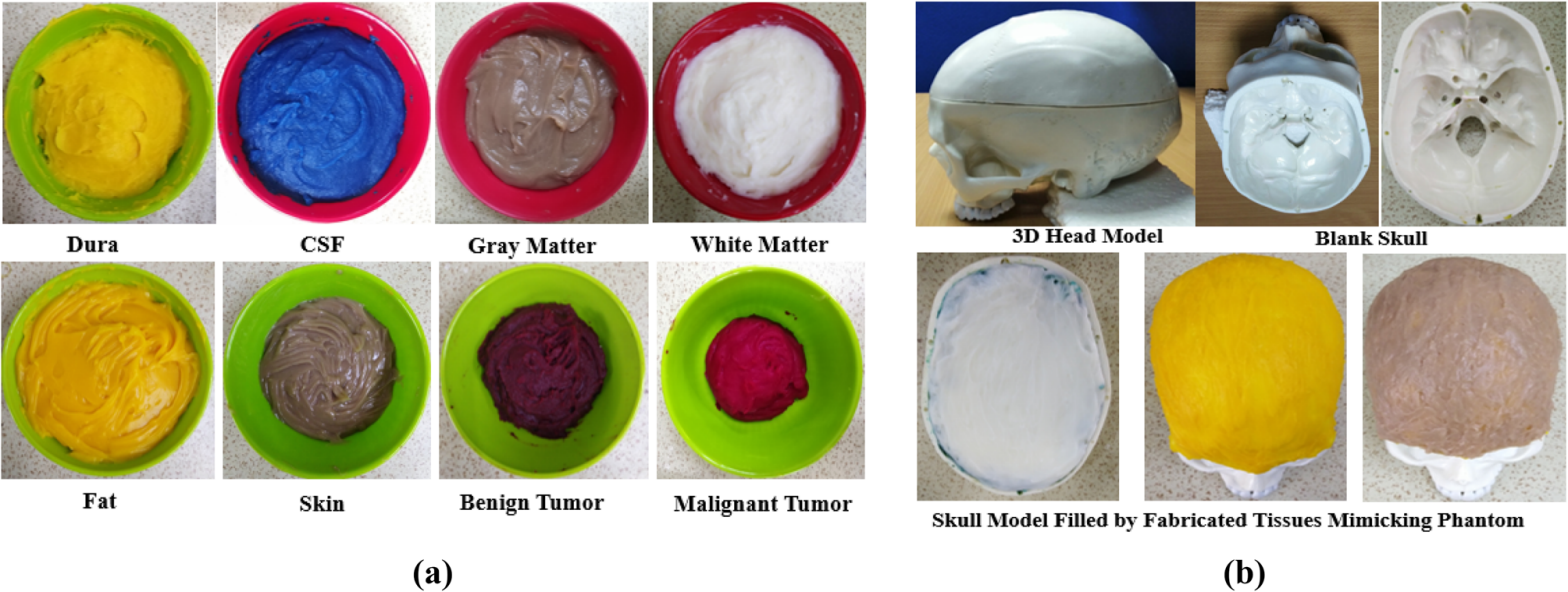
(**a**) Fabricated six tissue-mimicking phantom layers, including tumors; (**b**) 3D blank head model and skull model filled by fabricated tissue-mimicking phantom.

Initially, a six-layered (i.e., Dura, CSF, Grey Matter, White Matter, Fat, and Skin) tissue-mimicking phantom, benign, and malignant tumor tissues are fabricated. According to the presented recipe in^43^, the brain layers and tumors are fabricated. The dielectric properties of the tumors (i.e., benign and malignant) are considered as presented value in^44,45^. The benign tumor is fabricated as almost circular with a regular shape, whereas the malignant tumor is fabricated as an elliptical and triangular irregular shape^45^. The required ingredients for phantom fabrication are presented in Tables 4 and 5. The brain tissues of the human head are comprised with four layers: DURA, CSF, White Matter, and Gray Matter. The permittivity and conductivity of real human brain tissues^26^ are presented in Table 6. The permittivity and conductivity are changed with respect to the changing the frequency. The permittivity is gradually decreased with increasing the frequency. On the other hand, the conductivity is gradually increased with increasing the frequency. In this research, the permittivity and conductivity of brain tissues are considered as a reference of the human head model. The measured values of fabricated tissues can be 3 to 5% slightly increased or decreased due to ingredients tolerance. However, the permittivity and conductivity of brain tissues at 2 GHz are presented in Table 6. The fabricated phantoms are shown in Fig. 21a. Later, the tissue-mimicking phantoms and tumors are inserted layer by layer into the 3D head model shown in Fig. 21b.

**Table 4:** Ingredients for 500 gm phantom/tissues fabrication.

Ingredients	Water (gm/ml)	Corn flower (gm)	Gelatine (gm)	Agar (gm)	Sodium azide (gm)	Propylene glycol (gm)	Nacl (gm)	Sodium benzoate (gm)	N-propanol (gm)
Tissues									
DURA	361.90	120.65	0.00	4.58	1.80	9.65	1.20	0.00	0.00
CSF	418.75	10.15	0.00	56.20	1.85	7.45	5.60	0.00	0.00
Gary matter	403.25	82.95	0.00	5.20	1.75	4.60	2.30	0.00	0.00
White matter	353.35	134.30	7.05	0.00	1.75	3.55	0.00	0.00	0.00
Benign tumor	409.85	13.35	–	63.75	1.80	4.55	6.40	0.00	0.00
Malignant tumor	405.85	14.55	–	63.55	1.30	5.30	6.40	1.55	1.25

**Table 5:** Ingredients for 500 gm fat and skin fabrication.

Ingredients	Water (gm/ml)	Corn flower (gm)	Gelatine (gm)	Canola oil (gm)	Kerosene (gm)	Glycerin (gm)	Nacl (gm)	Sodium benzoate (gm)	N-propanol (gm)	TX-151
Tissues										
Fat	54.50	111.90	30	75.00	79.50	100	1.50	1.50	31.10	0.00
Skin	240.00	150.00	0.00	0.00	46.00	0.00	1.60	2.00	0.00	25

**Table 6:** Permittivity and conductivity of brain tissues of the human head and fabricated phantom.

Name of the brain tissues	Human head		Fabricated head phantom	
	Permittivity at 2 GHz	Conductivity at 2 GHz	Permittivity at 2 GHz	Conductivity at 2 GHz
Dura	52.12	1.23	47.93	1.66
CSF	68.64	2.413	63.78	2.71
Gray matter	52.73	0.942	47.74	1.38
White matter	38.89	0.591	35.98	0.765

**Figure 22:**
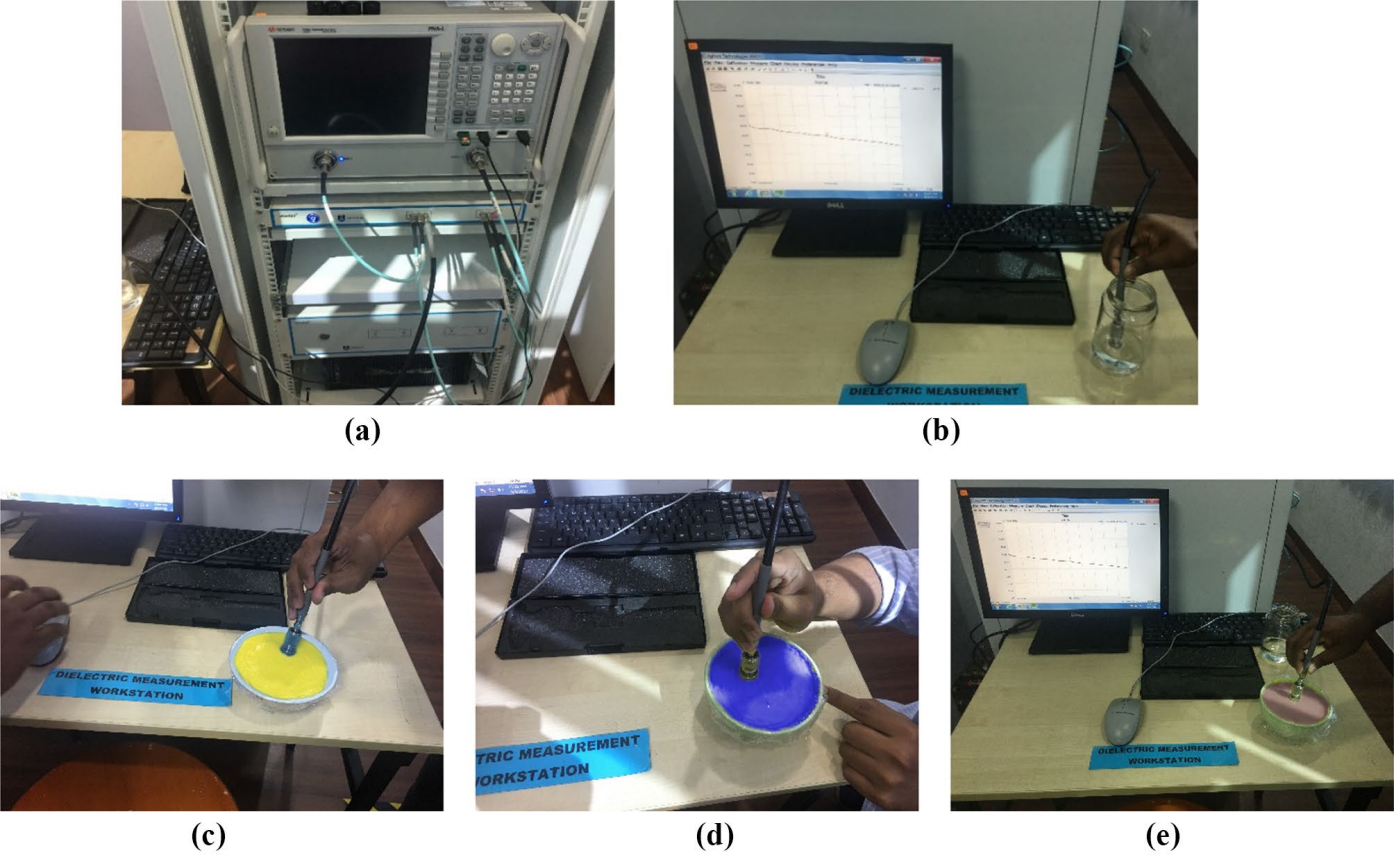
Phantom measurement process: (**a**) Phantom measurement setup with PNA; (**b**) Calibration with sterile water by open-ended coaxial probe; (**c**–**e**) Fabricated phantom measurement process using probe kit: DURA, CSF, and Gray Matter samples respectively.

**Figure 23:**
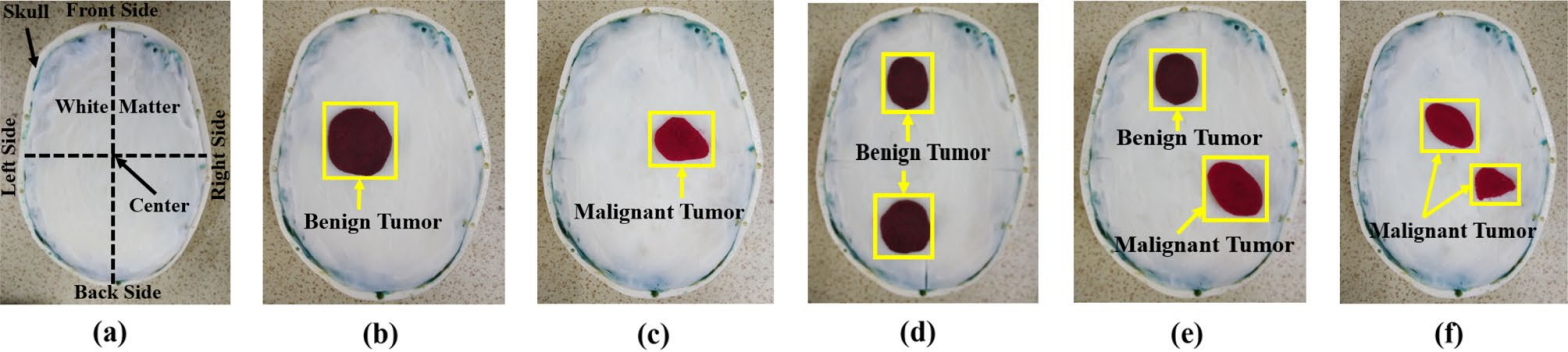
The fabricated phantom model includes tumors at different locations: (**a**) Skull model filled with fabricated tissues; (**b**) With a single benign tumor; (**c**) With a single malignant tumor; (**d**) With two benign tumors; (**e**) With a single benign and a single malignant tumor; (f) With two malignant tumors.

**Figure 24:**
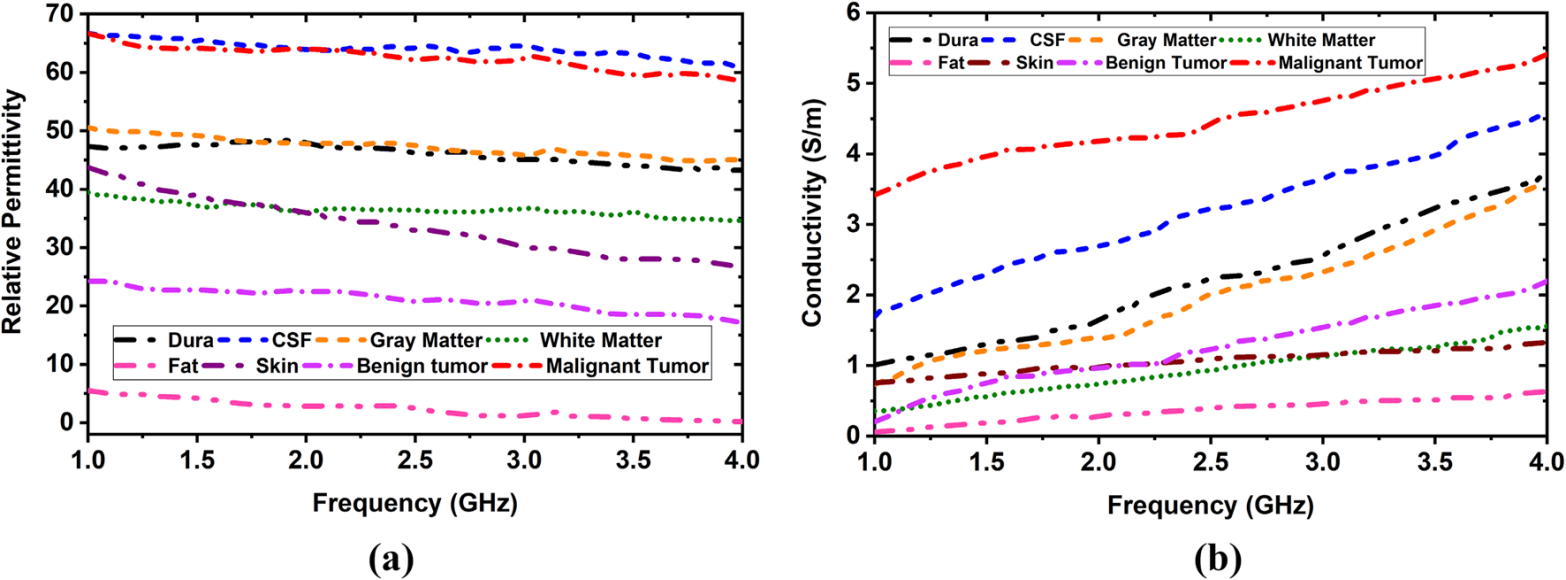
Measured results of dielectric properties of the fabricated tissues: (**a**) Relative permittivity; (**b**) Conductivity.

The dielectric properties of the fabricated tissues are measured by the dielectric probe kit KEYSIGHT 85070E and a power network analyzer (PNA, Model: PNA-L N5232A; 300 kHz to 20 GHz). The fabricated tissue-imitating head phantom's dielectric characteristics are measured using the open-ended coaxial probe technique. This method can be used for both in-vivo and ex-vivo measurements over a wide frequency range and is straightforward, non-destructive, and effective. However, the complicated heterogeneous structures and uneven surfaces in the homogenous structures are seen as limits for reliable measurements. The main limiting elements for measuring contents are calibration methods and measurement tools like PNA. For an accurate measurement, additional environmental aspects like temperature, humidity, and pressure as well as system elements like the cleanliness of the probe tip should be considered. The transmission line where the microwaves travel through the coaxial wire is cut off for the dielectric probe kit. Reflected signals are produced by the impedance mismatch between the probe and the targeted tissue sample, and these signals are then transformed into complicated permittivity values. Figure 22a,b depicts the initial calibration phase utilizing the VNA and coaxial probe with 25 cm3 of sterile water. Next, to guarantee enough contact between the sample and the coaxial probe while completing the measurements, each sample of the head phantom is sliced separately. The visual inspection of the inner and outer sections of the sample is reviewed to verify the consistency after the outside part of the sample is polished flat to ensure there is no gap between the coaxial probe and sample component. For collecting data with greater accuracy, the coaxial probe is positioned on the sample surface multiple times at random. The final assessment is then made by calculating the mean value from the many data points that were collected. The sample measurement setup picture of the fabricated phantom components is depicted in Fig. 22c–e. The benign and malignant tumors are inserted at different positions in the model to perform the measurement by the brain imaging system. The geometrical orientation of the head model filled with tissues is depicted in Fig. 23a. The benign and malignant tumors are inserted at different locations in the head model, which is depicted in Fig. 23b–f. The measured dielectric properties of the fabricated tissues are illustrated in Fig. 24.

### Microwave brain imaging system implementation and imaging results

**Figure 25:**
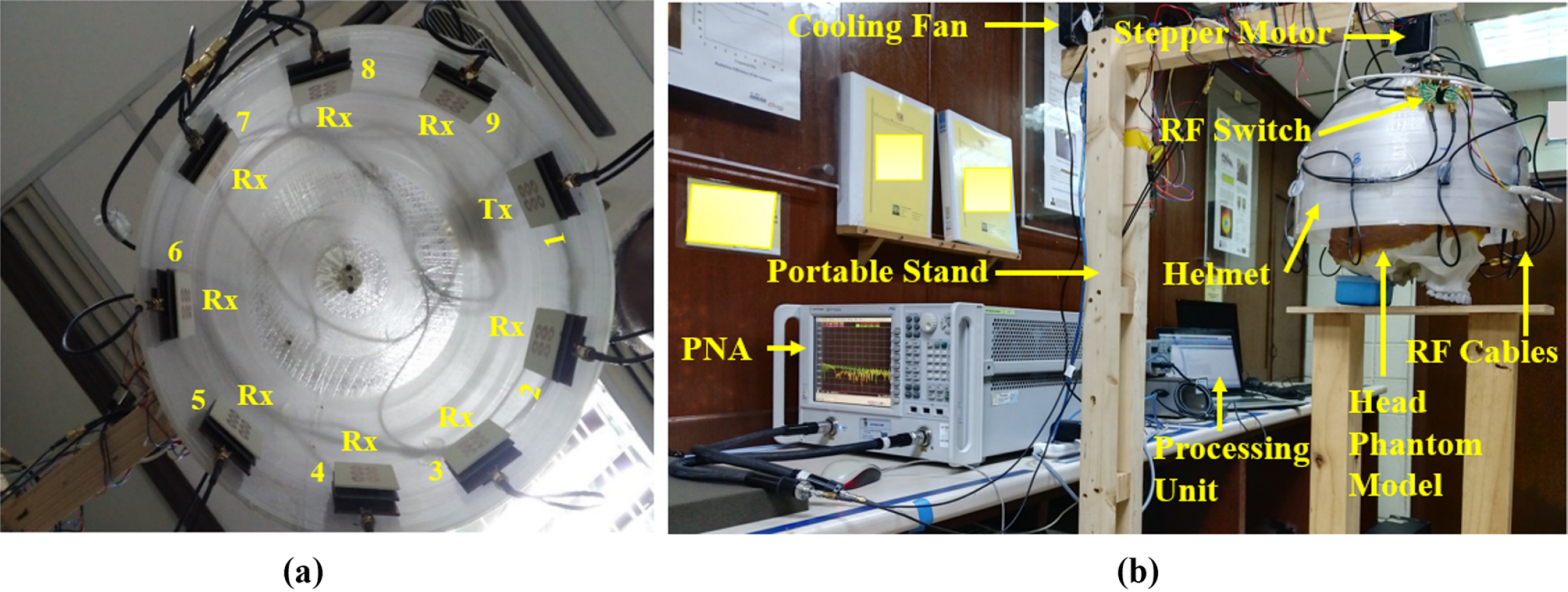
Implemented microwave brain imaging system: (**a**) Nine antenna array setup inside the helmet; (**b**) Overall imaging system.

The proposed microwave brain imaging (MBI) system is implemented to verify the performance. Figure 25 shows the overall implemented MBI system. The proposed system consists of an MTM-loaded 3D stacked nine antenna arrays, a custom-made half-cut elliptical-shaped helmet, a stepper motor, a portable stand, RF switch, microcontroller, and a PNA E8358A transceivers. The stepper motor is attached to the portable stand, which rotates clockwise with a 7.2° angle at every step to cover the whole (360°) area. The helmet is connected with the motor by the motor shaft. The diameter of the helmet is 250 mm. The antenna is attached inside the helmet by double-sided foam tape. The angular distance from antenna to antenna is 40° to cover whole are of the system. The antenna position is set 100 mm up from the bottom point of the helmet to adjust the phantom head position. The PNA is connected with the computer through the GPIB port, port A is connected with the transmitting antenna, and Port B is connected to RF switch for receiving the backscattered signals. The fabricated six-layered 3D head phantom model is placed at the center position of the helmet to verify the system performance. For verifying the outcomes of the system, the simulated and measurement imaging setup and their corresponding reconstructed brain images comparison are presented in Table 7. The Hugo head model is considered for simulation setup and fabricated phantoms with human 3D head skull model is considered for measurement purposes. The benign and malignant tumor(s) with different shapes are inserted in both head models and investigated the imaging performances. For better understanding, the cartesian coordinate system is used to present the geometrical view of the images for finding the localization of the tumor in the images. From the cartesian coordinate (x, y), it is easy to find the location of the tumor in the reconstructed images. The x-axis and y-axis are assumed in mm. For instances, in simulation Setup 2, the location of the benign tumor is (− 12, 12) whereas, in measurement Setup 2, the location of benign tumor is (− 4, 4). However, the tumor locations (i.e., benign and malignant) as a cartesian coordinate in reconstructed images for both simulation and measurement setup are summarized in Table 8.

**Table 7:**
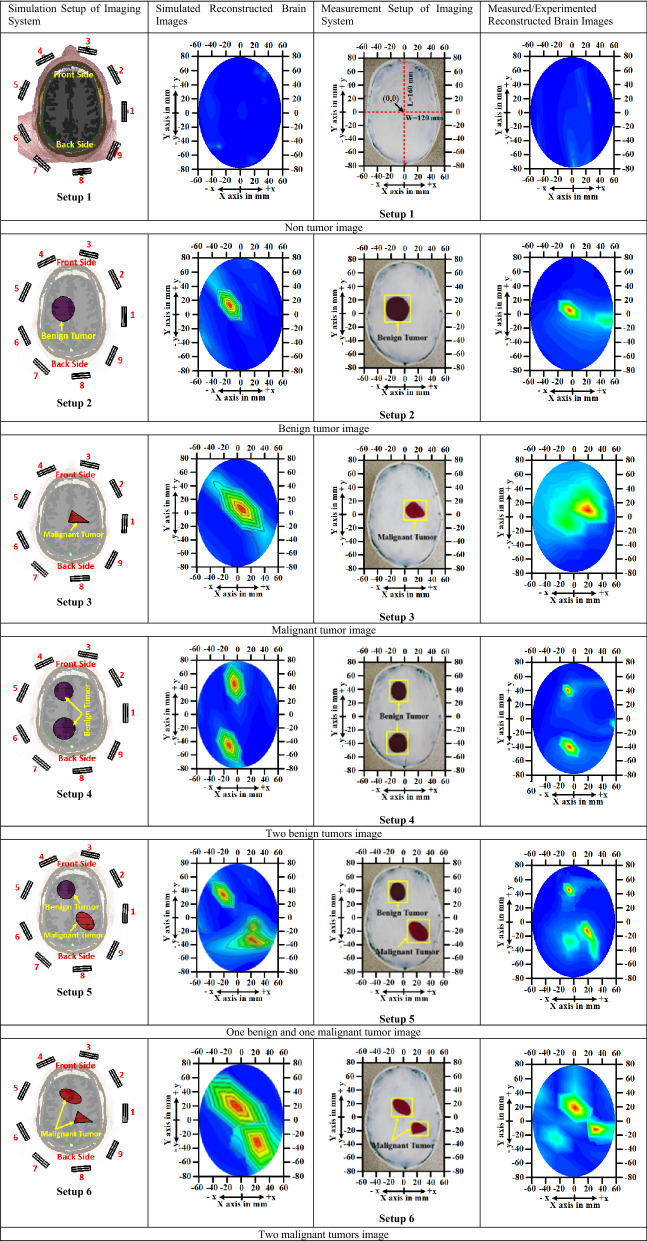
Comparison between simulation and measurement/experimental imaging setup with tumor localization.

**Table 8:** Summarized comparison results of tumor location as a cartesian coordinate in reconstructed images.

Simulation setup			Measurement setup		
Setup No	Tumor type	Tumor location in mm (x, y)	Setup No	Tumor type	Tumor location in mm (x, y)
1	No tumor	No location	1	No tumor	No location
2	Benign tumor	(− 12, 12)	2	Benign tumor	(− 4, 4)
3	Malignant tumor	(8, 4)	3	Malignant tumor	(20, 10)
4	Benign tumor 1	(− 6, 44)	4	Benign tumor 1	(− 8, 40)
	Benign tumor 2	(− 12, − 50)		Benign tumor 2	(− 8, − 46)
5	Benign tumor	(− 20, 30)	5	Benign tumor	(− 8, 44)
	Malignant tumor	(24, − 36)		Malignant tumor	(20, − 16)
6	Malignant 1	(− 8, 20)	6	Malignant 1	(0, 20)
	Malignant 2	(28, − 36)		Malignant 2	(30, − 12)

**Figure 26:**
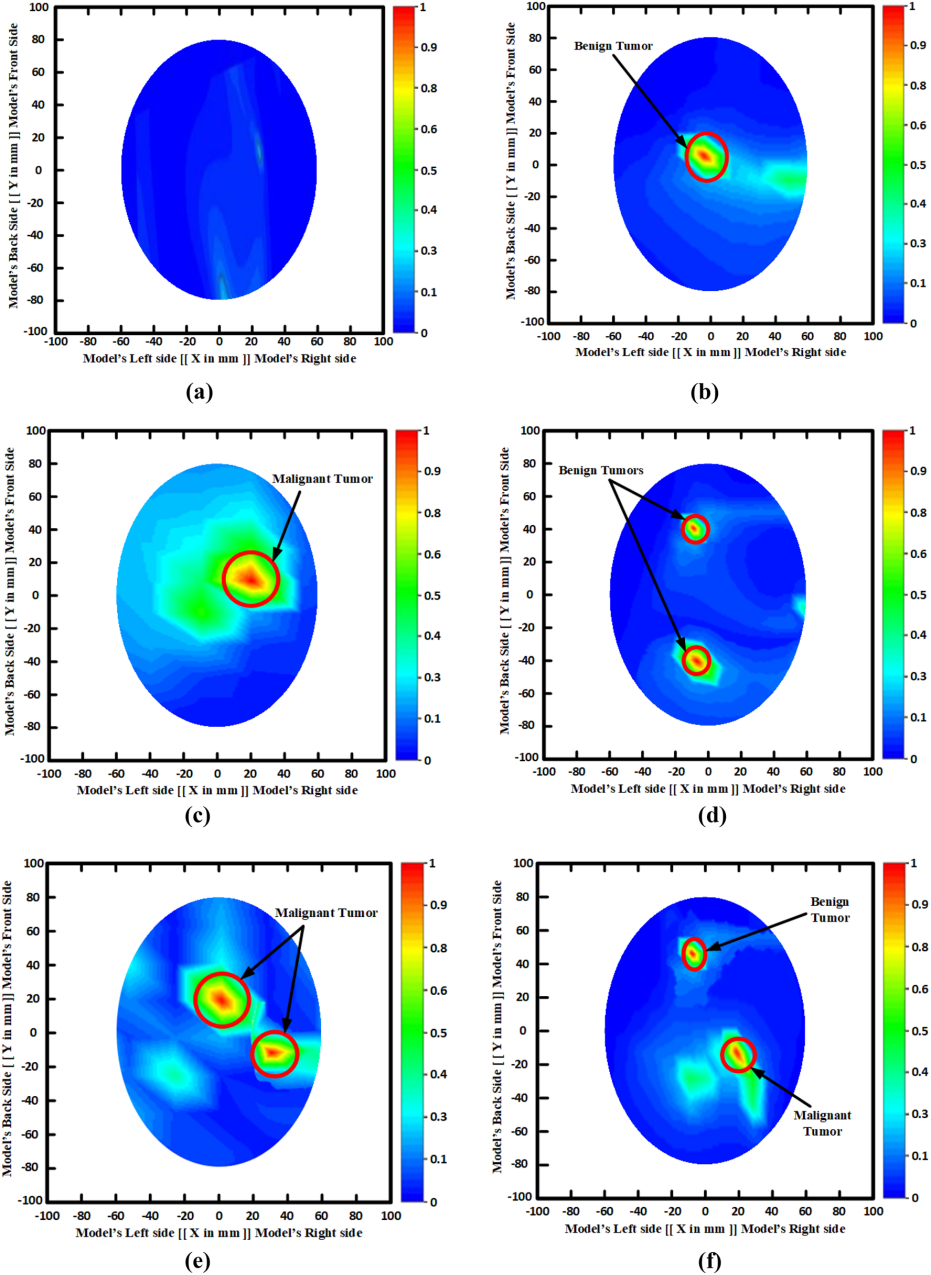
Brain tumor detection results: (**a**) Non-tumor; (**b**) With a single benign tumor; (**c**) With a single malignant tumor; (**d**) With two benign tumors; (**e**) With two malignant tumors; (**f**) With a single benign and a single malignant tumor.

Furthermore, the backscattered signals (i.e., S11, S21, S31, … S91) are collected by the PNA in each 7.2 rotation from the system. Later, the collected signals are post-processed and utilized by the Iteratively Corrected Coherence Factor Delay-Multiply-and-Sum (IC-CF-DMAS) image reconstruction algorithm^17^ to reconstruct the brain tumor images. This is the improvement of IC-DMAS algorithm and novelties of the used IC-CF-DMAS algorithm compared to other image reconstruction algorithm are (i) It has ability to produce noiseless image with a clear tumor object localization in reconstructed images, (ii) It can reconstruct images with more than one tumor object, and (iii) It takes less computational time to reconstruct brain images. Figure 26a–f represent the non-tumor image (i.e., healthy brain), single benign tumor image, single malignant tumor image, two benign tumors image, two malignant tumors image, and a single benign tumor and a single malignant tumor image, respectively. It is observed that the reconstructed images exhibited very low noises within the regional head area. The proposed MBI system is able to generate images of the brain with target tumors and location. The circular red mark in the images presents the tumor(s) detection and position. The left and bottom side axis labels are used to detect the location of the tumor as a cartesian coordinate. Eight different tumor location(s) are considered in this work to examine and evaluate the performance. It is concluded that the proposed implemented MBI system can identify and trace the brain tumor(s) with a location inside the brain that verifies system capability. Finally, the overall comparison of the proposed antenna with the state-of-the-art in terms of the antenna structure, dimension, substrate name, substrate layer, MTM inclusion, operating band, FBW, realized gain (Re. Gain), fidelity factor (FF), Maximum SAR (Max. SAR), imaging system, detected no. of tumor by the system, and used phantom model for testing is presented in Table 9.

**Table 9:** Overall observation and comparison of the proposed antenna with the state-of-the-art.

References	Antenna structure	Dimension (mm^3^)	Name of substrate	Layer	MTM inclusion	Operating band, FBW (%)	Re. Gain (dBi)	FF (%)	Max. SAR (W/kg)	Imaging system	Detected tumor	Tested phantom model
^20^	Antipodal Vivaldi	150 × 150 × 1.60	FR4	1	No	1.00–3.30, 106.97%	6.60	NP	NP	Single antenna, simulated	One	Semi realistic homogeneous
^21^	Microstrip	70 × 50 × 1.55	FR4	1	No	0.80–1.20, 40%	NP	NP	NP	Two antennas, Simulated	One	3D plastic head model
^22^	Microstrip	64 × 42 × 1.60	FR4	1	No	1.40–2.52, 57.14%	3.5	80	0.300	Nine antennas, simulated	One	Simulated Hugo head model
^24^	Microstrip	32 × 24 × 1.60	FR4	1	Yes	0.75–1.60, 72.345	4.5	NP	NP	Twelve antennas, simulated	One	3D SAM head phantom model
^25^	Microstrip	31.68 × 31 × 0.75	Rogers RO3003	1	No	6.30–7.40, 16.05%	6.57	NP	0.922	Single antenna, Simulated	One	Simulated head model
^26^	Microstrip	50 × 44 × 1.524	Rogers RO4350B	1	No	1.70–3.71, 74.30	5.65	98	0.0023	Twelve antennas, Simulated	One	Simulated Hugo head model
^27^	3D	40 × 25 × 10.5	Copper sheet	3	No	1.65–1.72, 4.10%	5.2		NP	Two antennas, Simulated	One	Simulated head model
^28^	3D	50 × 34 × 25.00	FR4	3	No	1.41–3.57, 87%	2.6	NP	NP	Single antenna, Experimental	One	3D SAM head phantom model
^29^	Conformal	25 × 28 × 5.60	Flexible	1	No	1.00–4.30, 124%	5.65	92	NP	Thirteen antennas, Experimental	One	Liquid head phantom model
^30^	3D	25 × 25 × 10.50	Copper sheet	3	No	2.65–2.91, 9.35%	6.60	85	NP	Two antennas, Simulated	One	Simulated head model
^16^	3D	53 × 22 × 21.575	Rogers RT5880	1	Yes	1.51–3.55, 80.75%	5.95	NP	NP	Nine antennas, experimental	Two (not mentioned)	Four layered tissue mimicking phantom
This work	3D stacked	50 × 40 × 8.67	Rogers RT5880 and RO4350B	3	Yes	1.37–3.16, 79.20%	6.67	98	0.0018	Nine antennas, experimental	Two (benign, malignant)	Six layered tissue mimicking phantom

*NP Not reported.

## Conclusion

A metamaterial (MTM) loaded three-dimensional (3D) stacked wideband antenna array is used in microwave brain imaging system to detect brain tumors inside the brain. A spider net-shaped metamaterial unit cell is designed and employed in the proposed antenna to enhance antenna performances. The antenna is fabricated on cost-effective Rogers substrate materials. The top layer and middle layer are fabricated on RT5880, and bottom layer is fabricated on RO4350B substrate material. The optimized dimension of the proposed antenna is 50 × 40 × 8.66 mm^3^ with an operating band of 1.37–3.16 GHz. The fabricated antenna achieved high radiation efficiency, gain with a high-fidelity factor. The fidelity factor is investigated for H-plane and E-plane as well as in four cases: SBS (X-axis and Y-axis), FTF, and BTB. The antenna showed a high-fidelity factor in the H-plane and in BTB cases. The specific absorption rate of the antenna is calculated with and without MTM and ensured satisfactory field penetration in the head tissue. The implemented system is investigated by utilizing a six-layered tissue-mimicking head phantom and reconstructed the brain images using IC-CF-DMAS algorithm to detect tumors. The benign and malignant tumors are effectively detected by the MBI system. Finally, it is decided that the proposed antenna can be a suitable candidate for the MBI system to successfully detect and locate benign and malignant tumors inside the brain.

## Data Availability

The datasets used and analyzed during the current study are available from the corresponding author on reasonable request.
